# The effect of individual and mixed rewards on diabetes management: A feasibility randomized controlled trial

**DOI:** 10.12688/wellcomeopenres.14824.3

**Published:** 2019-02-05

**Authors:** J. Jaime Miranda, María Lazo-Porras, Antonio Bernabe-Ortiz, M. Amalia Pesantes, Francisco Diez-Canseco, Socorro del Pilar Cornejo, Antonio J. Trujillo

**Affiliations:** 1 CRONICAS Center of Excellence in Chronic Diseases, Universidad Peruana Cayetano Heredia, Lima, Peru; 2 School of Medicine, Universidad Peruana Cayetano Heredia, Lima, Peru; 3 Department of Endocrinology, Hospital Nacional Arzobispo Loayza, Lima, Peru; 4 Department of International Health, Johns Hopkins Bloomberg School of Public Health, Baltimore, MD, USA

**Keywords:** behavioral economics, Diabetes, Diabetes management, Diabetes control, complex interventions, feasibility trial, Peru

## Abstract

**Background: **Incentives play a role in introducing health-related benefits, but no interventions using mixed incentives, i.e. a combination of individual and group incentives, have been tested in individuals with type 2 diabetes mellitus (T2DM). We evaluated the feasibility of implementing individual- and mixed-incentives, with and without a supportive partner, on glycated haemoglobin (HbA1c) control and weight loss among patients with T2DM.

**Methods:** This is a feasibility, sex-stratified, single-blinded, randomized controlled study in individuals with T2DM. All participants received diabetes education and tailored goal setting for weight and glycated haemoglobin (HbA1c). Participants were randomly assigned into three arms: individual incentives (Arm 1), mixed incentives-altruism (Arm 2), and mixed incentives-cooperation (Arm 3). Participants were accompanied by a diabetes educator every other week to monitor targets, and the intervention period lasted 3 months. The primary outcome was the change in HbA1c at 3 months from baseline. Weight and change body mass index (BMI) were considered as secondary outcomes.

**Results: **Out of 783 patients screened, a total of 54 participants, 18 per study arm, were enrolled and 44 (82%) completed the 3-month follow-up. Mean baseline HbA1c values were 8.5%, 7.9% and 8.2% in Arm 1, Arm 2, and Arm 3, respectively. At 3 months, participants in all three study arms showed reductions in HbA1c ranging from -0.9% in Arm 2 to -1.4% in Arm 1. Weight and BMI also showed reductions.

**Conclusions: **Individual and mixed cash incentives show important reductions in HbA1c, weight and BMI in patients with type 2 diabetes mellitus after 3 months.  Recruitment and uptake of the intervention were successfully accomplished demonstrating feasibility to conduct larger effectiveness studies to test individual and mixed economic incentives for diabetes management.

Registration: ClinicalTrials.gov Identifier NCT02891382

## Introduction

Evidence indicates that there are major benefits to be achieved by preventive care among patients with diabetes ^1– 4^, yet adherence to healthy behaviors and pharmacological treatment remains a challenge worldwide ^5– 8^. Non-monetary and monetary rewards may play a role in introducing health-related benefits by incentivizing behavior changes among individuals with type 2 diabetes mellitus ^9^. Whilst some have explored the effect of monetary incentives on certain behaviors, very few studies have evaluated the efficacy of cash rewards for patients with diabetes ^10^. One randomized controlled trial utilizing cash rewards in African American veterans with poor glycaemic control found that, at six months, the group assigned to cash rewards slightly improved HbA1c, with a non-significant mean reduction in HbA1c from 9.5% to 9.1% ^11^.

Most of the studies to date have considered an individual-based approach towards motivation and incentivization, but these have limitations ^12– 14^. Individual cash rewards appear to be more effective in the case of standardized activities or in cases when it is relatively easy to observe effort, to monitor outcomes, and when changes are driven by short-term behaviors and goals. In the same vein, individual cash rewards may be less effective in situations where i) highly complex tasks are needed; ii) in activities and target behaviours that require dealing within social norms, trust, and reputation; iii) in activities where it is difficult to clearly observe effort and thus change in outcomes; and iv) in activities that involve long term change ^12, 14^.

Behavioral economics theory, however, suggests that group incentives, or mixed rewards, could outperform individual incentives due to cooperation ^15, 16^. Cooperation refers to a supportive action that happens between two “equals” doing something to achieve a common goal or mutual benefit ^17^. Unlike “helping”, cooperation is an exchange between “equal” group members and it is opposed to competing and acting in a selfish manner ^18^. Altruism on the other hand is a helpful act “carried out in the absence of obvious tangible rewards for the helper ^17^.” Altruism lies at the opposite end of competition. Testing mixed or group-based approaches for diabetes prevention or management is relatively new, and some studies are being conducted ^10, 19^.

Owing to the limitations of individual-based incentivization strategies ^9^, this study was designed to evaluate the feasibility of implementing individual- and mixed-incentives, with and without a “supportive” partner, on glycated haemoglobin (HbA1c) control and weight loss among patients with type 2 diabetes mellitus. These interventions were anticipated to promote positive lifestyle changes, including knowledge about diabetes self-management, diet changes and increased physical activity. Additional exploratory analyses included modelling the change of continuous variables using repeated measurements over the study period; and, subgroup analyses according to the number of appointments completed (weekly over a 6-week period), and by early success in terms of number of payments provided in the first two appointments. Overall, this study was framed as a feasibility study designed to generate hypotheses to be tested in future larger studies and to explore the feasibility of conducting complex interventions for diabetes management in terms of fieldwork enrolment and follow-up, delivery and uptake of the intervention, and challenges of having a partner support.

## Methods

### Trial design

Feasibility trial, we followed the CONSORT 2010 statement extension for randomized pilot and feasibility trials ^20^. This study was a single-centre, sex-stratified, with balanced randomization [1:1:1], single-blinded, non-pharmacological, parallel arm randomized controlled study. The intervention period lasted 3 months. Protocol and CONSORT checklist are provided as Supplementary File 1 and Supplementary File 2.

### Formative research

Our formative research was oriented to improve understanding of the challenges of living with and managing type 2 diabetes mellitus, the opinion of diabetic patients about a cash reward program and the social support they have. We conducted (1) a questionnaire with 100 patients with diabetes from the same hospital as the trial was going to be conducted ^21^, and (2) 20 in-depth interviews among patients with similar characteristics ^22^. The questionnaires helped us determine a reasonable amount for the cash rewards, which ended up being 80 PEN if the participant lost one kilogram after two weeks, approximately 10% of minimum wage (Peru’s minimum salary wage for year 2017 was 850 PEN/~$264 USD), and 400 PEN (~$124 USD) if the HbA1c was reduced by 1% or more after 3 months. Through the qualitative interviews, we were also able to better understand the role of the family in diabetes management, which meant it was feasible to ask for a cooperative partner for the trial, and that most (83%) would choose their spouse or partner and 23% would choose their children. During this formative phase we also learned that 42% of participants had tried to lose weight at least once since their diagnosis of diabetes.

### Participants and setting

Patients attending the outpatient clinic of the endocrinology service from Hospital Nacional Arzobispo Loayza were approached and invited into the study from July to October 2016 (15 weeks). This hospital is located in Lima, Peru’s capital, and is one of the national tertiary hospitals from the Ministry of Health, serving low- to medium-income population. There were 783 people assessed for eligibility.

Subjects, irrespective of their HbA1c levels, (1) with a diagnosis of type 2 diabetes mellitus, (2) aged 18–70 years old, (3) a body mass index (BMI) 25-39.9 kg/m ^2^, (4) without diabetes-related complications, i.e. blindness, amputations, foot ulcers or being on dialysis, (5) not receiving pharmacotherapy for weight loss or corticosteroids, (6) not serving as companion/team support for another participant in the trial, and (7) with the ability to provide informed consent, were considered eligible for the study. Exclusion criteria were pregnancy status and having a diagnosis of cancer or other serious comorbidity.

To be eligible as a partner for the participant with diabetes, individuals were to be 18–70 years old, available and committed to supporting the participant in achieving their goals during the intervention, without a physical or mental impairment that prevented helping the participant to improve their disease management, and able to provide informed consent.

### Fieldwork procedures

Fieldworkers explained the study to potential participants. If the patient was eligible, the fieldworker explained the procedures and intervention, and gave a copy of the informed consent form, and asked him/her to come back with their potential partner to explain and invite them to join the study. Once they returned, written informed consent was obtained from the two of them. If the participant was at the recruitment facility with their potential partner, the fieldworkers explained the study to both and also applied the informed consent to the partner. Participants and their partners were reimbursed a fixed amount for their transportation costs to attend the appointments.

After recruitment, participants were interviewed by a trained fieldworker to complete a baseline questionnaire ( Supplementary File 3), including information related to socio-demographic information, co-morbidities, characteristics about diabetes care, perception of diabetes ^23^, and depression symptoms (Patients Health Questionnaire [PHQ-9]). Additionally, information from the participant’s partner was also collected, including socio-demographics, physical activity and willingness to help their partner. During the appointments with the diabetes educator, the participants’ weight, knowledge about diabetes self-management ^24^, diet, and physical activity were recorded.

At the end of the intervention the same baseline questionnaire was applied. Also, all participants that had a companion support answered five questions assessing the perceived role they felt their partner played in improving their diet, increasing physical activity, and overall diabetes management and weight-loss intervention goals.

HbA1c was measured using high-performance liquid chromatography (D10, BioRad, Munich, Germany), traceable to the Diabetes Control and Complications Trial reference study as certified by the National Glycohemoglobin Standardization Program. All samples were analyzed in a single facility, and, for quality assurance, the quality of assays was checked with regular external standards and internal duplicate assays and monitored by BioRad.

Upon completion of the trial, 29 individuals (17 participants and 12 partners) were selected for in-depth interviews focused on their experience participating in this cash rewards program. For this, we aimed to select 6 participants per study arm (18 in total), and 6 partners per study arm (12 in total), and we managed to interview 29/30 out of the planned target. Within each group of 6, we selected 3 individuals (patients or their partners) who “performed well during the intervention”, defined as those who were able to lose 3Kg or more, and 3 others who did not. All interviews were transcribed verbatim and entered into qualitative analysis software (ATLAS.ti 8.0, Scientific Software Development GmbH, Berlin, Germany) using a predefined set of codes developed from the themes of the interview guide, which addressed three aspects of the intervention: i) perspective regarding the economic incentive; ii) perspective regarding diabetes education received; and iii) the perspective regarding the support received by his chosen companion for the intervention (only for participants randomized to arms 2 and 3). On the other hand, the interview guides elaborated for the companions addressed two aspects of the intervention: i) perspective of the companions regarding the economic incentive; and ii) perspective regarding the support provided to the participant. Data from each code was then organized in matrices and summaries for each code were produced as well as an identification of the key quotes given by participants.

### Intervention

This study explored the role of cash rewards, with or without a “supportive” partner, in changing the behavior of individuals with type 2 diabetes mellitus, specifically by promoting healthy lifestyles through tailored nutritional advice, delivered by a diabetes educator, attached to individual goal-setting with cash rewards. Both actions were expected to contribute to achieve weight and HbA1c targets. All participants received diabetes education and tailored goal setting for weight and HbA1c. Participants were randomly assigned into three different intervention arms: Arm 1, individual incentives, i.e. the cash rewards for the patient if the goals were achieved; Arm 2, mixed incentives-altruism, patient had a partner but cash rewards were for the patient; and, Arm 3, mixed incentives-cooperation, where participants had a partner but the cash was given to both the patient and the partner in a ratio of 50%-50%.

***Diabetes education.*** Diabetes education was provided by a nutritionist with previous experience in weight management for people with diabetes. In the introductory meeting, the diabetes educator explained to the participants all the procedures, including details of the number of sessions, and the amount of money they will receive if they met the target weight. Depending on the study arm allocation, the diabetes educator also explained whether the participant needed a partner and whether the money was going to be only given to the participant or to both, the participant and the partner. Participants received a manual with information about diabetes management with a tailored weight loss plan and the bi-weekly goals. Each participant was offered up to seven follow-up sessions with the diabetes educator, every 2 weeks, plus a final session, thus totaling up to nine one-to-one interactions during the trial, provided that the participant attended all of their meetings. One section of the manual included a logbook to register their challenges regarding introducing changes in diet and physical activity, as well as any questions they would like to ask the diabetes educator in the following session. The information from this logbook provided the starting point for conversations with the diabetes educator during the follow-up sessions, every 2 weeks. Recruitment of participants was done in a staggered manner to ensure the diabetes educator did not have a high concentration of patients in one single week. Thus, there was a close coordination between the fieldworker scheduling the first appointment and the diabetes educator who was already seeing participants on a bi-weekly basis, by sharing a Google Docs spreadsheet were both aware of the slots available.

***Goal setting.*** To determine eligibility for receiving a cash reward, three goals were pre-specified: (1) Weight loss, 80 PEN ($25 USD) if the participant lost one kilogram over a period of two weeks. These goals were reset based on the most recent weight result, (2) HbA1c level, 200 PEN ($62 USD) if the participant achieved, at the end of the study, a decrease of <1% compared to their baseline level, and (3) HbA1c level and control, 400 PEN ($124 USD) if the participant achieved, at the end of the study, a decrease ≥1% of A1c or reached levels of A1c ≤6.5% compared to their baseline level. At the end of the study, targets for weight loss and HbA1c were evaluated independently, i.e. participants could receive more than one reward provided that each independent target was achieved.

***Partner support.*** In Arms 2 and 3 of the study, each participant had a partner that received information about diabetes care in the introductory meeting, and a brochure to guide and support the treatment process of the participant. The activities of the partner were recorded in the case logs bi-weekly, and they were offered to join the follow-up sessions with the patient, every 2 weeks, but these were not compulsory. It was only compulsory to attend the first and at last follow-up session.

***Recipient of the cash rewards.*** Two strategies were considered as to who would be the recipients of the rewards. In Arm 1 (individual) and Arm 2 (mixed-altruism) the reward was provided to the participant. It was up to the participant to share (or not) the reward with their partner. In contrast, in Arm 3 (mixed-cooperation) the reward had to be shared, i.e. the participant and their partner received 50% of the cash reward each.

We kept the size of the reward equal between the treatment groups to avoid an income effect at the household level, i.e. the reward size was the same across all study arms regardless of the involvement of the partner support.

### Outcomes

***Primary and secondary outcomes.*** The primary outcome was the change in HbA1c at 3 months from baseline. Weight and change body mass index (BMI) were considered as secondary outcomes, specifically the change at 3 months from baseline.

***Intermediate outcomes.*** The intervention were anticipated to promote changes in three indicators, namely knowledge about diabetes self-management, diet, and physical activity.

***Risk factors.***The profile of risk factors were obtained from responses to the following questions: Alcohol: In the last year, how often did you drink alcohol beverages? (Never vs. ≤1/month, 2-4/month, 2-3/week or >4/week). Smoking: Do you smoke at least one cigarette per day? (Yes vs. No). Walking: During the week, how often do you walk at least 30 min? (>5 days, 4-5 days, 2-3 days vs. ≤1 day). Fruit intake: How often you eat fruit? One portion of fruit is one fresh fruit, or a glass of juice made out of fresh fruit, or 1 cup of fresh fruit chopped (>3, 2-3, 1 vs. <1 portions per day). Vegetable intake: How often do you eat vegetables? One portion of vegetables equals one cup with vegetables in any presentation or preparation: crude, cooked, steamed, stir fried, or grilled (>3, 2-3, 1 vs. <1 portions per day).

### Sample size

Being a feasibility study, a formal sample size calculation was not performed. The researchers aimed to enroll 54 participants, 18 participants per study arm, over a 3-month period because it was considered this would be a large enough sample to gather information about the practicalities of delivering incentive-oriented diabetes self-management strategies that would impact HbA1c in patients with type 2 diabetes mellitus. As such, the successful completion of this study would inform about recruitment, uptake, and attrition rates as well as engagement with the intervention.

### Randomization

Randomization was conducted as per CONSORT criteria ^20^. All participants were randomly assigned (1:1:1) to receive one of the three interventions using a computer-generated list of numbers. Randomization was stratified by sex (female:male ratio of 2:1), and within each stratum, the randomization process involved blocking with block size of 6. For allocation concealment, participants were randomized using sequentially numbered, opaque, sealed envelopes. The sealed envelope was assigned after the patient had been recruited and all baseline measurements were completed. The random allocation sequence was generated by one researcher. This researcher was not involved in the fieldwork activities of the trial. A fieldworker was responsible of participants’ recruitment and taking baseline information. The diabetes educator was not involved in the randomization process. The diabetes educator was responsible for weighing participants and to provide the cash rewards to participants, the participants and the diabetes educator were not blind to the intervention. Physicians, other care providers and researchers were blinded to the study group.

### Analytical methods

All analyses were conducted with STATA V.13.0 (StataCorp, College Station, TX, USA). Descriptive analyses, describing means and frequencies were conducted. Following trial recommendations, no formal statistical testing for comparison of baseline data was pursued ^25, 26^. Differences in HbA1c, weight, and BMI were estimated using 3-month and baseline information, and comparisons were conducted using t-paired test within the same study arm, and Student’s t-test between arms using the Arm 1 as the reference. Analysis of intermediate outcomes and subgroups according to the number of appointment completed (split into two group using the median) and the number of payments provided in the first two appointments (0, 1, and 2) were conducted using the same tests. Finally, linear mixed models with a random intercept were used to assess changes of weight, BMI, and diabetes self-management values as continuous variables using the information captured every 2 weeks.

### Ethical approval

The study protocol was reviewed and approved by the ethics committee from the Hospital Nacional Arzobispo Loayza, in Lima, Peru. The formative research received ethical approval from both the Universidad Peruana Cayetano Heredia and Hospital Nacional Arzobispo Loayza.

## Results

### Participants characteristics

**Figure 1.**
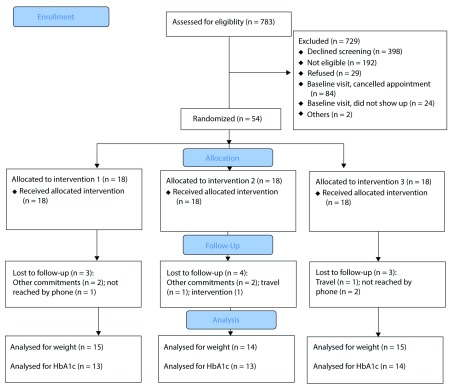
Flowchart of study participants.

A total of 783 participants were screened, and 54, 18 per study arm, were enrolled. Of them, 44 (82%) completed the 3-month follow-up ( Figure 1). The baseline demographic and clinical characteristics for each study group are shown in Table 1. The participants’ mean age was 55 years, 36 (67%) were females, and half of all participants reported being employed. The majority of participant’s family income was <$500 USD per month, and the participants were not the major contributors to it.

**Table 1.** Baseline demographic and clinical characteristics.

	Arm 1 Individual (n=18)	Arm 2 Mixed Altruism (n=18)	Arm 3 Mixed Cooperation (n=18)
Sociodemographic profile			
Female	12/18 (67%)	12/18 (67%)	12/18 (67%)
Age (years), mean ± SD	54.7 ± 9.8	55.5 ± 9.8	54.2 ± 7.6
Education			
Primary level or lower	4/18 (22%)	3/18 (17%)	2/18 (11%)
Primary to secondary level	5/18 (28%)	10/18 (56%)	8/18 (44%)
Secondary level or higher	9/18 (50%)	5/18 (28%)	8/18 (44%)
Occupation			
Employed	8/18 (44%)	9/18 (50%)	9/18 (50%)
Unemployed	5/18 (28%)	2/18 (11%)	2/18 (11%)
Housewife	5/18 (28%)	7/18 (39%)	7/18 (39%)
Monthly income ^†^			
<850 PEN (<$264 USD)	3/16 (19%)	5/16 (31%)	6/15 (40%)
850-1,500 PEN ($264-$465 USD)	10/16 (62%)	7/16 (44%)	4/15 (27%)
>1,500 PEN (>$465 USD)	3/16 (19%)	4/16 (25%)	5/15 (33%)
Highest contribution to household’s income (yes)	8/18 (44%)	5/18 (28%)	6/18 (33%)
Living with a partner (yes)	11/18 (61%)	10/18 (56%)	15/18 (83%)
Household size (number of people), mean ± SD	5.4 ± 4.2	4.2 ± 2.1	4.8 ± 2.1
Diabetes-related profile			
Time of diabetes diagnosis (years), mean ± SD	6.6 ± 5.8	7.4 ± 8.4	5.9 ± 3.8
Self-reported health (very good, good, fair) *	14/15 (93%)	16/17 (94%)	13/18 (72%)
Had HbA1c measured in the last 3 months (yes)	11/17 (65%)	11/16 (69%)	12/18 (67%)
Pharmacological treatment			
Oral hypoglycemic drugs	12/17 (71%)	11/17 (64%)	17/18 (94%)
Insulin	1/17 (6%)	3/17 (18%)	0/18 (0%)
Both	4/17 (23%)	3/17 (18%)	1/18 (6%)
Has diabetes complications			
Eye problems	11/18 (61%)	13/18 (72%)	13/18 (72%)
Foot ulcers	2/18 (11%)	2/18 (11%)	0/18 (0%)
Renal problems or dialysis	5/18 (28%)	3/18 (17%)	3/18 (17%)
Health-related profile			
Comorbidities			
Hypertension	5/18 (28%)	5/18 (28%)	4/18 (22%)
Stroke	1/18 (6%)	2/18 (11%)	1/18 (6%)
Depression (PHQ-9 >14)	0/18 (0%)	1/18 (6%)	2/18 (11%)
Lifestyle and behavioral risk factors			
Alcohol consumption (never, last 12 months)	8/18 (44%)	7/18 (39%)	7/18 (39%)
Cigarette smoking (yes, at least one per day)	2/18 (11%)	1/18 (6%)	3/18 (17%)
Walking 30 min (≤1 day per week)	7/18 (39%)	6/18 (33%)	5/18 (28%)
Fruit intake (<1 portion per day)	8/18 (44%)	3/18 (17%)	2/18 (11%)
Vegetable intake (<1 portion per day)	4/18 (22%)	3/18 (17%)	3/18 (17%)

All values presented correspond to n (%), unless otherwise stated. ^†^Peru minimum salary wage for year 2017 was 850 PEN, the category used for monthly income. Average exchange rate for year 2017 was 1 PEN = 0.31 USD. *Self-reported health, the corresponding figures (not shown) belong to poor or very poor categories.

The household’s average size was around 5 people, and more than half of participants lived with a partner. The average duration of the diagnosis of diabetes was 6.6 years, and large proportions indicated fair to very good levels of self-reported health. Most patients with diabetes were on oral drugs, some already report foot and renal diabetes complications, and hypertension was the most common comorbidity reported.

There were 36 participant partners, with a mean age of 44.9 years (SD 16.8); 61.1% were female. The role of partners was fulfilled by a spouse (33.3%), offspring (33.3%), friends (13.9%), siblings (5.6%), and the remaining (13.9%) were other relatives such as mother, grandchildren, cousin or father.

### Primary and secondary outcomes at 3 months

Mean baseline HbA1c values were 8.5%, 7.9% and 8.2% in Arm 1, Arm 2 and Arm 3, respectively. At 3 months and relative to their baseline levels, participants in all three study arms showed reductions in HbA1c ranging from -0.9 in Arm 2 to -1.4 in Arm 1 ( Table 2). Weight and BMI also showed reductions, and these were more pronounced in Arm 1.

**Table 2.** Primary and secondary outcomes at baseline and 3-month results, by study arms.

	Arm 1 Individual	Arm 2 Mixed Altruism	Arm 3 Mixed Cooperation	Arm 2 vs. Arm 1 (p-value)	Arm 3 vs. Arm 1 (p-value)
Primary outcome					
HbA1c (%)					
Baseline	8.5 ± 2.2	7.9 ± 3.1	8.2 ± 2.1	0.99	0.99
3 months	6.9 ± 1.7	7.2 ± 1.6	7.1 ± 1.7	0.99	0.99
Δ (3-mo vs baseline)	**-1.4 ± 1.4**	**-0.9 ± 1.2**	**-1.1 ± 1.6**	0.05	0.05
Secondary outcomes					
Weight (Kg)					
Baseline	76.9 ± 10.7	84.5 ± 17.4	85.1 ± 22.3	0.59	0.48
3 months	72.6 ± 11.0	82.2 ± 17.2	84.7 ± 22.7	0.45	0.20
Δ (3-mo vs baseline)	**-2.9 ± 2.2**	-0.4 ± 2.5	-0.4 ± 3.0	0.04	0.03
BMI (Kg/m ^2^)					
Baseline	33.1 ± 4.8	34.6 ± 6.0	36.1 ± 10.1	0.99	0.67
3 months	31.6 ± 5.2	33.8 ± 5.7	36.0 ± 9.8	0.99	0.31
Δ (3-mo vs baseline)	**-1.2 ± 0.9**	-0.2 ± 1.0	-0.2 ± 1.4	0.05	0.05

Values in bold are those significant (p<0.05) when comparing difference between 3-month and baseline assessment (within each arm).

### Intermediary outcomes at 3 months

There was evidence of effect of the intervention on diabetes self-management, diet and physical activity when comparing 3-month and baseline assessments, especially in Arm 1 ( Supplementary File 4-Supplementary Table 1).

### Subgroup analysis

Data from all study participants were pooled to generate subgroups according to the number of appointments completed and by early success in terms of number of payments provided in the first two appointments ( Table 3).

**Table 3.** Primary and secondary outcomes at baseline and 3-month results, by study’s subgroups.

	Subgroups by number of appointments completed			Subgroups by number of payments provided in the first two appointments			
	Lower	Higher	p-value *	0	1	2	p-value *
	N = 24	N = 30		N = 24	N = 21	N = 9	
Primary outcome							
HbA1c (%)							
Baseline	8.0 ± 3.0	8.3 ± 2.0	0.66	7.9 ± 3.1	9.0 ± 1.9	7.1 ± 1.1	0.11
3 months	6.8 ± 1.3	7.1 ± 1.7	0.64	7.5 ± 2.1	7.3 ± 1.3	6.0 ± 0.4	0.09
Δ (3-mo vs baseline)	-0.8 ± 1.5	**-1.2 ± 1.4**	0.49	-0.6 ± 1.6	**-1.8 ± 1.3**	**-1.0 ± 0.9**	0.07
Secondary outcomes							
Weight (Kg)							
Baseline	85.2 ± 16.9	79.7 ± 17.9	0.26	78.3 ± 16.8	83.4 ± 11.8	89.6 ± 27.6	0.23
3 months	84.2 ± 19.2	77.7 ± 17.4	0.28	74.8 ± 15.7	82.7 ± 13.9	85.1 ± 26.9	0.27
Δ (3-mo vs baseline)	0.3 ± 1.9	**-2.0 ± 2.8**	0.009	0.6 ± 1.9	**-1.6 ± 2.1**	**-4.5 ± 2.3**	<0.001
BMI (Kg/m ^2^)							
Baseline	35.5 ± 6.4	33.9 ± 8.0	0.42	33.5 ± 6.0	33.8 ± 4.2	39.3 ± 13.2	0.10
3 months	35.5 ± 6.7	33.0 ± 7.5	0.29	32.4 ± 5.1	33.4 ± 5.1	37.3 ± 12.6	0.25
Δ (3-mo vs baseline)	0.1 ± 0.8	**-0.9 ± 1.2**	0.01	0.2 ± 0.8	**-0.7 ± 0.8**	**-2.0 ± 1.1**	<0.001

Values in bold are those significant (p<0.05) when comparing difference between 3-month and baseline assessment (within each arm). * p-values in the columns are for comparisons between subgroups.

In terms of the primary outcome, those who completed a higher number of appointments had a significant reduction of 1.2% in HbA1c levels at 3 months. On the other hand, those participants who were successful in receiving at least one cash reward in the first two appointments were more likely to achieve reductions in HbA1c, nearly 1 to 2 units lower compared to their baseline levels.

Weight showed more marked reductions as per subgroups analysis. Those who completed a higher number of appointments had, on average, reductions of up to 2 kg, compared to those in the lower range of appointments completed, who only showed reductions in the order of 0.3 kg relative to their baseline values. In addition, those who were highly successful securing two cash rewards in the first two visits had an average reduction of 4.5 kg, three times higher than those who only received one cash reward, and these two groups had more marked reductions in weight than those who did not receive a cash reward in their first two appointments.

### Exploratory analysis: change in indicators every 2 weeks

Participants in Arm 1 showed gradual changes in the desired direction of benefit in all of these indicators. Weight reductions of 1 kg were observed at 2 weeks, reductions of up to 2 kg between week 4 to week 8, and >2 kg by week 10. Diabetes self-management scores also increased, almost doubling towards the end of study’s intervention period ( Supplementary File 4-Supplementary Table 2).

No changes in diabetes self-management scores were noted in Arms 2 or 3. In these arms, only changes in weight and BMI were observed, but these became evident only after 10 weeks (Arm 2) and 6 weeks (Arm 3) from baseline. Changes in weight achieved towards the end of the study period were in the order of reductions of 2.3-2.4 Kg, equivalent to reductions of ~1 unit of BMI.

### The partner support

Of the 27 participants that answered the final questionnaire and had partner support, 23 (85%) of participants assessed that their partners’ support was “a lot” or “fair” towards achieving an improved diet. Similar results, “a lot” or “fair”, were also reported for increasing their physical activity (20/27, 74%), adhering to their medication regime (23/27, 85%), overall diabetes management (23/27, 85%), and achieving weight-loss goals (23/27, 85%).

When asked to explain these answers, participants said that their supportive partners motivated them either for taking care of their diet or for doing exercise. Other partners reminded patients of their medication, prepared diabetic-appropriate meals, went on walks together, accompanied to their appointments or told them what they could or could not eat. Overall, participants felt supported by their partners.

In the in-depth interviews, most participants stated that they liked that they were given the freedom to choose the partner and that their choice was based on the high level of trust in that person, which, in turn, enabled a more constant interaction between them. Such interactions revolved around talking about the recommendations provided by the diabetes educator and the strategies to put them into practice. Having a partner involved also facilitated the engagement with other family members who were informed about the disease and the intervention.

On the other hand, interviewed partners stated that the intervention helped them improve their support to the person with diabetes by increasing their (partner) knowledge about the disease and having more tools to help patients lose weight and manage the disease.

### Harms

No harms or unintended effects in each group were observed.

## Discussion

This study was designed to evaluate the feasibility of implementing individual and mixed cash rewards on HbA1c and weight loss among patients with type 2 diabetes mellitus. After completion of the 3-month intervention, notable reductions were observed in HbA1c, weight and BMI, and also in some of the intermediate and self-reported outcomes. Subgroup analysis confirm reductions in weight and BMI among those who complete a higher number of appointments and more clearly among those who achieve early success, i.e. those who were eligible to receive a cash reward during their first two appointments.

Contrasting with the effect of oral antidiabetic drugs, which lowers HbA1c in the order of 0.5%-1.25% ^27^, our results, derived from a complex intervention relying on diabetes education with tailored goal-setting with or without group (partner) support, showed reductions in HbA1c of similar magnitude to those achieved with pharmacological treatment. This study was intended to generate hypotheses, and the rationale for using the term feasibility in this work was informed by a combination of issues related to the fieldwork, to packaging and deploying the intervention, and the experience of the support partner. In terms of fieldwork, we document the ability to approach, identify eligible individuals, as well as to recruit, enroll, and follow-up sufficient number of patients and partners. Key aspects of the intervention include the capacity to deliver the intervention, an intense intervention with tailoring features, and to have a separate team to measure outcomes, expressed in the results reported. Acceptability of the intervention and challenges of the implementation were also captured in the post-study interviews reporting the experience of the partner support. The accomplishment of recruitment and conduction of this study, together with the uptake of the intervention by the study participants as well as the lessons from the experience of the partner support, demonstrate the feasibility of conducting larger effectiveness studies using individual incentives and mixed incentives involving carers for supporting diabetes management.

Our study contributes with ongoing debates about the challenges of lifestyle modification, a cornerstone for diabetes management and control ^7, 28, 29^. This study provides pragmatic insights into two of the top-ten research priorities established by people living with diabetes and their carers, i.e. “what is the best way to encourage people with type 2 diabetes mellitus, whoever they are and wherever they live, to self-manage their condition, and how should it be delivered?” and “how can people with type 2 diabetes mellitus be supported to make lifestyle changes to help them to manage their condition, how effective are these lifestyle changes, and what stops them from working?” ^30^ The participation of companion partners in our study elucidated that although participants assessed that the support was important, the provision of family support for people with chronic conditions is not free of problems. As described elsewhere, families can sometimes undermine lifestyle changes or self-management when they have a poor understanding of the disease or when their “supportive style” is perceived as nagging by patients ^22^.

Mixing social support with cash rewards aims to overcome the limitations of the individual cash rewards approaches. For example, sustaining complex tasks may place the individual under pressure or feel negatively motivated because of the burden associated with the management of diabetes ^31– 34^. In this sense, our mixed incentives arms tried to explore different concepts. Arm 2 was purely based on altruism, and, in a way, the reward was similar to Arm 1, the individual incentives. On the contrary, Arm 3 was specifically designed to test cooperation and the effort directly compensated both parties. Interestingly, when designing a complex intervention, and as shown by our results, engaging participants with earlier “gains” such receiving a cash reward during their first two appointments, and receiving a higher dose of the intervention expressed by the completion of a higher number of appointments signal to improvements HbA1c, between 1 and 2% lower, and weight and BMI at 3 months. This observation is in line with the behavioral literature that has identified that small but tangible rewards, delivered with high frequency, can aid engagement with complex tasks ^9^.

In minority groups from high-income settings, culturally appropriate interventions appear to have a more pronounced effect in reducing HbA1c levels compared to usual care, reported in the order of reductions of 0.5 units at 3 months ^35^. Our study showed much higher, even doubling, effect sizes over the same period of time, thus indicating promising scenarios for further HbA1c control. Our pilot was not designed to formally test comparisons between study arms, yet the results obtained were in the expected direction and improvements were observed after the completion of the 3-month study period in all primary and secondary outcomes, both within study arms and between arms. Also, the rationale for the selection of the main outcomes was based in that they can be objectively ascertained and are integral part of diabetes management ^6, 28^. We express caution with the interpretation of results as it cannot be ascertained whether the changes in primary or secondary outcomes are due to the effect of the intervention in a given study arm. If anything, changes were observed in reducing HbA1c levels and important lessons were obtained for the practicalities of conducting larger studies using mixed incentives and enabling activities between patients, companion supports and diabetes educators.

Various systematic reviews have covered the effect of incentives and cash rewards on lifestyle behaviours ^36– 39^, but none of these reviews evaluated its impact on type 2 diabetes mellitus. Other trials have explored or are exploring the effect of individual versus group financial incentives for reducing weight loss in high-income settings, with rewards up to $520 USD ^19, 40^, much larger than the ones provided in our study. In the UK, Relton *et al*. ^41^ assessed an NHS-commissioned financial incentive weight loss program, aiming to reduce between 6.8 to 22.7 kg in 3 to 7 months, with incentives ranging from £70 to £425. They found that 40% achieved clinically significant weight loss ^41^. Whilst there is not direct comparability between the type or intensity of intervention, in our study we expand upon these experiences by showing the feasibility of reducing levels of HbA1c.

One of the strengths of the study is the ability to accommodate an incentive-based strategy together with an effective goal-setting approach for patients with uncontrolled diabetes in low-resource settings and observing benefits in objective indicators such as HbA1c and weight reductions. Also, our formative phase explored and guided the characterization of the size of the cash reward, a detail usually missing in the cash incentive literature ^38, 42^. The introduction of two different mixed-incentives arms affords a pragmatic understanding that tackling diabetes management is a task that can be absorbed and accommodated by both, patients and their companion support, and thus expanding the arsenal of strategies to deal with diabetes. In our study, all participants were exposed to diabetes education and goal setting strategies. Whilst some of the effects are expected to be accrued through self-determination and opportunities to deal with the burden of diabetes’ self-management, there is an element where face-to-face consultations are also needed to maintain engagement and motivation ^43^, and as such, this element will need to be maintained in larger studies.

Reward-based strategies delivered with goal setting and diabetes education can be considered an intensive intervention. Yet, the status quo of diabetes control, with almost half of UK and US adults with diabetes as well as more than 90% of Peruvian patients not meeting the recommended goals for diabetes care ^44– 46^, deserves innovative responses. Also, there are recent promising results arising from the DIRECT study, conducted in the primary care network, which has shown that diabetes remission can be achieved through intense 12-month weight loss management program ^4, 47^. The intensity of an intervention is one of many factors to consider. If weight-loss intense strategies can direct towards people engaging with better diet and lifestyles for longer periods, a lesser use of pharmacological medications with its associated risks, and even direct towards diabetes remission, then the scenario will be different. We will be able to complement this evidence, in a future study, by studying the effect of individual and group-based rewards.

Amongst the limitations are the difficulty in measuring and monitoring, bi-weekly, diet and physical activity. Whilst we used proxies for this measurements, it is clear that introducing such close monitoring of lifestyle habits may introduce additional challenges and increase burden among study participants ^34^, hence the preference for objective markers to monitor such as weight or HbA1c, which can be expanded to quality of life or utilization of health services if needed. Being a pilot study, a short-term strategy was pursued, requiring longer study intervention periods, and importantly, even longer follow-up periods without the intervention to study if the changes are maintained after the removal of the strategy. This is very important as we do not envisage to maintain participants under a program of incentivization for the long term, rather, this program is the medium to install and maintain the changes required for a successful diabetes management. Future studies should convey larger follow-up periods, capture meaningful patient-important outcomes ^48– 50^, explore whether the effect is sustained after removing the intervention of interest, and even consider testing whether mixed incentives are as effective or even better than individual-based incentives.

## Conclusions

After 3 months, our individual and mixed cash incentives program show important reductions in HbA1c, weight and BMI among patients with type 2 diabetes mellitus from low-income areas. Recruitment and uptake of the intervention were successfully accomplished, and attrition rates were not major hurdles, demonstrating feasibility of establishing larger efforts to expand the test individual or mixed monetary incentives for diabetes management.

## Data availability

Figshare: REDEEM Study. Feasibility pilot study. https://doi.org/10.6084/m9.figshare.7180802.v2 ^51^

Data are available under the terms of the Creative Commons Attribution 4.0 International license (CC-BY 4.0).

Transcripts of the in-depth interviews are not provided alongside this article because they contain sensitive and personal information, and they were recorded in Spanish language. However, these transcripts can be requested at no charge from the corresponding author. Please, should you want to access these data, send an e-mail to the corresponding author; the only condition of access is that we request you explain what you intend to do with these data.

## Supplementary material

Supplementary File 1: Study protocol (in Spanish).

Supplementary File 2: Completed CONSORT checklist.

Supplementary File 3: Questionnaires (in Spanish).
