# Supplementary material for: The effect of individual and mixed rewards on diabetes management: A feasibility randomized controlled trial
Source: Wellcome Open Res. 2019 Feb 5;3:139. Originally published 2018 Oct 31. [Version 3] doi: 10.12688/wellcomeopenres.14824.3 (PMC6325609; doi:10.12688/wellcomeopenres.14824.3)
Supplement: Supplementary file 1 [file wellcomeopenres-3-16478-s0000.tgz › 37223e94-17fe-4cfc-9eb0-70ea9307f62e_Supplementary_File_1_Cronicas_Protocolo_Incentivos_Parte_2_Abril_sin_anexos.pdf]

PROTOCOLO DE ESTUDIO

El efecto de incentivos individuales y mixtos en control de la diabetes: Parte 2

Patrocinador:

Medical Research Council (MRC), United Kingdom

Versión 1.0

Abril 2016

TABLA DE CONTENIDOS

EQUIPO DE TRABAJO 4
RESPONSABLES DEL ESTUDIO 5
LUGARES DE ESTUDIO.......... 6
RESUMEN DEL PROTOCOLO.......... 7
1. INTRODUCCION 8
1.1. Prevención de la diabetes 8
1.2. Incentivos en Salud 9
1.3. Diabetes e Incentivos Económicos 10
1.4. ¿Los incentivos económicos funcionan en América Latina? 11
1.5. Justificación 12
2. OBJETIVOS DEL ESTUDIO 14
Objetivo General: 14
Objetivos Específicos 14
3. METODOLOGIA DEL ESTUDIO 15
3.1. Diseño del estudio 15
3.2. Lugar de estudio 15
3.3. Población y criterios de selección 15
3.3.1Criterios de inclusión del paciente 15
3.3.2Criterios de inclusión del acompañante 15
3.3.3 Criterios de exclusión 16
3.4.Tamaño de muestra 16
3.5.Intervención 16
3.5 Resultados 19
3.6 Proceso de reclutamiento 19
3.6.1 Aleatorización y otros procesos 19
3.6.2 Procedimientos.......... 19
Fase 3: Seguimiento de pérdida de peso 20
Brazo1: Incentivos Individuales.......... 20
Brazo 2: Incentivos Mixtos: Establecido por Investigadores.......... 21
Brazo 3: Incentivos Mixtos: Establecido por Participantes 22
Brazo 3: Incentivos Mixtos: Establecido por participantes.......... 22
Fase 3: Seguimiento de experiencias con el estudio 23
3.7. Comunicación de resultados a pacientes 24
3.8. Cronograma 24
4. PROTECCION DE SUJETOS HUMANOS 25
4.7. Ética 25
4.8. Consentimiento Informado 25
4.9. Beneficios 25
4.10. Riesgos26
4.11. Pago a participantes 26
4.12. Confidencialidad 26

5. MANEJO DE DATOS Y PLAN DE ANÁLISIS 27
5.7. Control de calidad y seguridad de los datos 27
5.8. Plan de análisis 27
5.9. Cumplimiento del protocolo 28
5.10. Plan de seguridad de datos 28
6. REFERENCIAS BIBLIOGRAFICAS 29

EQUIPO DE TRABAJO

EQUIPO NACIONAL:

Jaime Miranda, MD, PhD

Investigador Principal
Profesor Investigador, Facultad de Medicina UPCH
Email: Jaime.miranda@upch.pe

Antonio Bernabé-Ortiz, MD, MPH

Investigador en Enfermedades Crónicas
Universidad Peruana Cayetano Heredia
Email: Antonio.Bernabe@upch.pe

Francisco Diez Canseco, BA, MPH

Investigador asociado a CRONICAS
Email: fdiezcanseco@upch.pe

M. Amalia Pesantes, MA, MPH, PhD

Postdoctoral Fellow
Email: maria.pesantes.v@upch.pe

EQUIPO INTERNACIONAL:

Antonio J. Trujillo, PhD

Co-PI

Director, Master of Health Science in Health Economics, Department of International Health
Associate Professor
Email: atrujil1@jhu.edu

Katherine Sacksteder, PhD

Associate, Johns Hopkins School of Public Health
Email: ksacksteder@yahoo.com

RESPONSABLES DEL ESTUDIO

Para consultas, contactar a:

(1) Dr. Jaime Miranda

Envíe un correo electrónico a jaime.miranda@upch.pe, o llame al +511-241-6978.

(2) Dra. M. Amalia Pesantes

Envíe un correo electrónico a maria.pesantes.v@upch.pe, o llame al +511-241-6978.

LUGARES DE ESTUDIO

**Nombre del lugar de estudio**	**:**	Hospital Nacional Arzobispo Loayza
**Dirección**	**:**	Avenida Alfonso Ugarte 848
**Investigador Responsable**	**:**	Dra. Socorro del Pilar Cornejo
**Teléfono**	**:**	6144646, Anexo 4300

RESUMEN DEL PROTOCOLO

Este estudio tiene por objetivo comparar el impacto que un incentivo económico tiene para mejorar el control de la hemoglobina glicosilada en pacientes con diabetes mellitus tipo 2, dependiendo si el esfuerzo se hace de manera individual o con el apoyo de alguien (familiar, amigo).

El uso de incentivos económicos para promover cambios de comportamiento, que persistan en el tiempo, han sido probadas en otros países de la región. Se han usado para cambiar hábitos como fumar y para bajar de peso, sin embargo no hay estudios que permitan conocer el impacto de dicha estrategia en el manejo de la diabetes mellitus tipo 2. Tampoco existen estrategias claras para determinar el monto del incentivo económico a utilizar para promover cambios de comportamiento (adherencia al tratamiento, incremento en actividad física y una dieta saludable) en los pacientes con diabetes mellitus tipo 2. También existen interrogantes con respecto a la frecuencia de los incentivos, el tipo de incentivo (efectivo u otro tipo de premio), la forma de pago, etc.

El presente estudio es de carácter exploratorio pues está orientado a documentar el proceso de implementación de una intervención orientada a una reducción en el peso y la hemoglobina glicosilada en pacientes con diabetes mellitus tipo 2 que reciben un incentivo económico en tres modalidades: (1) individual (sólo al paciente recibe el incentivo), (2) mixto predeterminado (el paciente y a la persona que lo ayuda a lograr su meta reciben el incentivo y lo dividen de acuerdo a lo establecido por el protocolo de investigación), y (3) mixto negociado (el paciente y a la persona que lo ayuda a lograr su meta reciben el incentivo y lo dividen luego de negociar entre ellos la proporción que le corresponde a cada uno). El incentivo económico será acompañando por educación en diabetes.

Al finalizar el estudio piloto, se usarán métodos cualitativos para documentar las experiencias y opiniones de los participantes en el estudio sobre la pertinencia del uso de incentivos económicos para promover hábitos asociados al manejo de la diabetes mellitus.

1. INTRODUCCION

El presente estudio es la segunda parte de un estudio exploratorio que permitirán luego, desarrollar un estudio clínico aleatorizado para identificar el impacto del uso de incentivos económicos en pacientes con diabetes mellitus tipo 2 para un mejor manejo de su hemoglobina glicosilada (HbA1c). La diabetes es una enfermedad crónica que presenta un enorme reto para los sistemas de salud en todo el mundo. Las prácticas para manejar la diabetes tales como los cambios en el estilo de vida, el aumento de ejercicio, y las modificaciones en la dieta han demostrado ser una forma efectiva de reducir las consecuencias y los costos de la enfermedad a largo plazo. Aunque sabemos que estas prácticas son efectivas, los pacientes con diabetes no suelen adherirse a ellas para controlar su enfermedad. Por ejemplo, en el Perú, el 70% de los pacientes con diabetes tienen un mal control glicémico y sólo el 3,3% cumple con la definición óptima de acuerdo a las recomendaciones de la American Diabetes Association (ADA), es decir con adecuado control de la glicemia, colesterol LDL y presión arterial. [1] En la población general, los valores obtenidos de control glicémico son igualmente pobres. Sólo el 7% de la población cuenta con un adecuado tratamiento y control de la glicemia (midiendo la glucosa en ayunas) y si los criterios son más estrictos (hemoglobina glicosilada o HbA1c <7%) el 0% de la población tiene un adecuado control de su enfermedad. [2] El mal manejo de una enfermedad crónica como la diabetes se asocia a un alto riesgo de complicaciones crónicas y comorbilidades, elevando los costos de salud asociados.

1.1. Prevención de la diabetes

Los ensayos clínicos en los países desarrollados y los estudios basados en la población han reportado beneficios de la atención preventiva para el control de la hemoglobina glicosilada en los pacientes con diabetes.[3] Las estrategias preventivas enfocadas en el manejo de la diabetes pueden añadir hasta cuatro años de esperanza de vida a una persona de 50 años, reducir la morbilidad relacionada con la diabetes, y aumentar considerablemente el bienestar.[4] A pesar del consenso médico sobre la eficacia de las medidas preventivas, la adhesión de los pacientes a los medicamentos en todo el mundo es bastante baja. En un estudio realizado en siete ciudades de América Latina en sujetos con diabetes tipo 2, el 78% de los individuos eran conscientes de su condición, el 33% recibía tratamiento, y sólo el 5% había alcanzado los objetivos terapéuticos.[5] En un estudio realizado en Lima, encontramos resultados similares: el 71% eran conscientes de su enfermedad, el 40% recibía tratamiento y sólo el 7% habían alcanzado los objetivos terapéuticos.[2]

Los bajos niveles de adherencia pueden ser explicados por factores de comportamiento del paciente con diabetes que lo impiden hacer lo que es mejor para su salud. Factores como falta de control o la poca capacidad de proyección hacia futuro podrían contribuir a este bajo nivel de prevención; por encima de los factores tradicionales como el acceso a los servicios de salud y la falta de recursos económicos para pagar por la prevención. En este contexto, el uso de incentivos económicos puros y mixtos podría ayudar con los problemas que enfrenta el paciente para hacer lo que más le conviene para cuidar su salud.

1.2. Incentivos en Salud

Los factores que le impiden a la persona con diabetes ver los beneficios de cuidarse podrían cambiar si es que se le ofrece un premio a la persona con diabetes que le dé un beneficio presente y que lo ayude a lidiar con los esfuerzos diarios de cuidarse. La literatura sobre incentivos sugiere que el efecto de incentivos para modificar el comportamiento depende de varios factores interconectados.[6] El pago de incentivos monetarios basados en el cumplimiento de determinadas medidas de prevención reduce el costo del manejo de la enfermedad, sin embargo, puede terminar por desplazar la motivación individual interna, clave del autocuidado de un paciente con una enfermedad crónica. Tanto el diseño como el modo de entrega de los incentivos pueden afectar el éxito de la intervención.

La literatura existente no permite afirmar si los incentivos monetarios promueven un cambio de comportamiento duradero y hay muchas dudas sobre su pertinencia para el manejo de enfermedades crónicas.[7] Las recompensas monetarias pueden ayudar a la formación de hábitos en el corto plazo ya que los beneficios de la prevención se acumulan durante mayores períodos de tiempo. La prevención de hoy puede llevar a disminuir el esfuerzo de prevención mañana, y los incentivos monetarios pueden ayudar a consolidar este proceso inicial.[8] Sin embargo, las recompensas en efectivo pueden no ser lo suficientemente grandes como para impulsar los cambios iniciales, especialmente una vez que los efectos iniciales del esfuerzo se disipan. También se puede argüir que los incentivos monetarios pueden ser eficaces en premiar resultados concretos gratificantes pero menos efectiva en premiar procesos como chequear los niveles de azúcar en la sangre todos los días o hacer ejercicio.

La literatura científica conductual establece que los incentivos entregados a los individuos deberían ser: frecuentes y en valores pequeños, ser positivos y no negativos, y promover la conexión entre pequeñas y grandes metas a largo plazo.[9-11] La evidencia acerca del impacto de los incentivos está en aumento pues hay varias intervenciones que buscan demostrar la utilidad de

usar incentivos económicos para la adherencia al tratamiento por abuso de sustancias,[12] y para promover la pérdida de peso y dejar de fumar.[13,14] Más recientemente, se han realizado estudios sobre el efecto de los incentivos en la donación de sangre, donación de órganos, el tratamiento del VIH y otras conductas pro-sociales.[15,16-17]

Por otro lado, existen comportamientos en los cuales no es fácil de medir el esfuerzo que ha realizado el paciente para lograrlos. En este caso, los incentivos económicos podrían no ser efectivos, este es el caso de la pérdida de peso pues es posible que dos personas pierdan la misma cantidad de peso en el mismo tiempo, pero el esfuerzo para lograr dicha meta probablemente fue diferente. Algunas revisiones indican que los incentivos económicos son efectivos en el corto plazo para la atención preventiva y en intervenciones conductuales bien definidas; además, existe evidencia suficiente para afirmar que los incentivos económicos son efectivos para el intercambio de estilo de vida a largo plazo requerida para la promoción de la salud. [18]

Poco se sabe sobre el efecto de los incentivos a nivel de individuo cuando dichos incentivos son entregados a los pares, a un familiar o amigo que proporciona apoyo en este proceso. Estos incentivos se denominan "incentivos mixtos" (*mixed incentives*) también pueden influir positivamente en el comportamiento individual, ya que utilizan el sentimiento de conexión con los demás y la responsabilidad en apoyarse mutuamente.

1.3. Diabetes e Incentivos Económicos

Se han realizado muy pocos estudios que evalúen la eficacia de los incentivos económicos en pacientes con diabetes y los impactos de esta enfermedad. Un ensayo clínico aleatorizado, con un tamaño muestral pequeño realizado en Estados Unidos, usó incentivos económicos en veteranos afroamericanos con mal control glicémico y encontró que el incentivo mejoró ligeramente los resultados de HbA1c en 1 a 2% en 6 meses; sin embargo, el resultado no fue estadísticamente significativo.[20] Otros estudios han demostrado el rol de los incentivos económicos en la estimulación y mantenimiento en personas que querían perder peso. Mientras que un estudio encontró que los incentivos económicos en formato de lotería podían facilitar la pérdida de peso comparado contra un grupo control, el efecto no se mantuvo tras la retirada del incentivo.[21] No obstante, en otro estudio en el que los incentivos estaban vinculados con el éxito del grupo, los participantes en el grupo que recibía el incentivo no sólo perdieron más peso, sino también mantuvieron la pérdida de peso mejor que el grupo control.[21] Estos resultados son interesantes, y sugieren que el estudio de los incentivos en el manejo de la diabetes requiere de mayor investigación.

1.4. ¿Los incentivos económicos funcionan en América Latina?

Las transferencias monetarias condicionadas (CCT en inglés por Condicional Cash Transfer) se han convertido en un método muy utilizado por los gobiernos como una estrategia para tratar de reducir la pobreza, mejorar la educación, y otros aspectos generales de desarrollo (como nutrición infantil), particularmente en los países de América Latina.[8] Varios países Latinoamericanos cuentan con algún programa de CCT, como México, Honduras, Nicaragua, Brasil, Colombia y Perú. Recientes evaluaciones sobre el impacto de estos programas, muestran que los indicadores de salud están mejorando como resultado de estos programas. [22] Es importante aclarar que la mayoría de resultados son producto de indicadores de proceso y directamente relacionados con el uso de los servicios de salud.

El primer programa de CCT implementado en América Latina fue “Oportunidades” en México, que incluyó el requisito de que los niños y las mujeres embarazadas tomen suplementos nutricionales y asistan a ciertos servicios de salud. Una evaluación del programa encontró asociación entre un mayor número de transferencias recibidas y resultados significativamente mejores en muchas áreas, incluyendo la memoria a corto y largo plazo, la integración visual y el desarrollo del lenguaje.[23]

“Atención a Crisis”, un CCT en Nicaragua, está condicionada al hecho de que las familias que reciben el dinero deben asegurar que los niños en edad pre-escolar asistan a sus citas de salud de forma regular y reciban la vacunación y micronutrientes/suplementos alimenticios cuando sea necesario. La evaluación de este programa encontró mejoras significativas en los resultados de salud. Curiosamente, estas mejorías se mantuvieron incluso dos años después del cese del programa y las transferencias de efectivo.[24] “Juntos”, el CCT en el Perú requiere que las mujeres embarazadas y madres de niños menores de 5 años usen ciertos servicios de salud con el objetivo de reducir la pobreza y aumentar la demanda de servicios públicos a cambio de cumplir con ciertas responsabilidades relacionadas con la salud y educación. Las familias beneficiarias de “Juntos” reciben una transferencia monetaria de 200 Nuevos Soles cada dos meses. “Juntos” es un programa relativamente nuevo y no existen muchos estudios publicados describiendo su impacto. Un reporte del Banco Mundial encontró que el consumo general de las familias aumentó un 33% y los niveles de pobreza se redujeron en 14%.[24] Además, encontró que Juntos aumentó la probabilidad de que los niños de los hogares beneficiados cumplan sus controles de Crecimiento y Desarrollo (CRED), que busquen ayuda profesional en caso de cualquier enfermedad, y la probabilidad de que las mujeres embarazadas dieran a luz en un establecimiento de salud, con un profesional experto.

Para entender mejor el impacto del componente en efectivo de CCT, es importante reconocer que estos programas aprovechan las sólidas estructuras familiares en América Latina, y potencian la responsabilidad de sus miembros para la mejora de la salud. La persona encargada de que los requisitos se cumplan no sólo se anima a ser responsable debido a una recompensa individual, sino para mejorar la situación de la familia. Esto remarca el potencial de utilizar el capital social y la responsabilidad para diseñar políticas más eficaces, y será un elemento importante en nuestro enfoque. Como se ha demostrado en los programas de CCT, el uso de incentivos económicos tiene el potencial de lograr efectos a largo plazo que duran más allá de la duración del programa de incentivos, lo que lleva a un mayor impacto en la salud pública.

Una investigación reciente analiza los factores que explican el manejo individual (*self management*) de la enfermedad de personas con diabetes e hipertensión usando bases de datos transversales para cinco países de América Latina.[25] Los hallazgos sugieren que el nivel de ingresos es el componente más importante para reducir las diferencias en el impacto de las prácticas preventivas. En los cinco países del estudio, el papel de los ingresos en el auto manejo de la diabetes en los pacientes diabéticos fluctúa entre el 23% en Chile a 58% en Argentina. Es interesante notar que el acceso servicios de salud (disponibilidad de seguros) [26] y el conocimiento sobre el auto manejo de la diabetes tiene un impacto mucho menor que los ingresos. Todos estos resultados sugieren que el pago en efectivo puede tener un rol importante en el cambio del comportamiento del individuo en el caso de los pacientes con diabetes.

1.5. Justificación

Los ensayos clínicos en países desarrollados y los estudios poblacionales indican que hay muchos beneficios del cuidado preventivo en pacientes con diabetes.[3, 27] A pesar del consenso médico sobre la eficacia de las medidas preventivas, mundialmente es difícil lograr los objetivos de control en pacientes con diabetes. Los datos disponibles enfatizan claramente la necesidad de un estudio que investigue los métodos que se necesitan para la prevención de la diabetes actualmente, y por qué se necesita específicamente en América Latina y otros sitios con escasos recursos.

A nivel mundial se han realizado muy pocos estudios que evalúen la eficacia de los incentivos en diabetes. En esta aplicación, se pretende explorar el papel de los incentivos para cambiar el comportamiento de las personas con diabetes tipo 2. El ahorro de costos o intervenciones costo-efectivas pueden evitar el impacto económico de las complicaciones diabéticas a largo plazo, tales como retinopatía, nefropatía, neuropatía y enfermedades cardiovasculares, así como las complicaciones a corto plazo, como las hospitalizaciones por mal control glucémico.[28] En nuestro estudio

aplicaremos los incentivos económicos para promover el cambio de comportamiento de nuestros pacientes para mejorar su control glicémico.

2. OBJETIVOS DEL ESTUDIO

Los resultados de este estudio serán usados para diseñar un ensayo clínico aleatorizado orientado a evaluar el impacto del uso de incentivos monetarios en pacientes con diabetes mellitus tipo 2. Para ello primero tenemos que responder las dos preguntas de investigación que precisamos a continuación.

Objetivo General:

Comparar los procesos de implementación de tres intervenciones que usan incentivos económicos para promover cambios de comportamiento en pacientes con diabetes mellitus tipo 2 a fin de identificar barreras y facilitadores vinculados al proceso de implementación de cada una de las intervenciones.

Objetivos Específicos

- Documentar la experiencia de los pacientes en el estudio de incentivos económicos.
- Documentar la experiencia de los acompañantes a los pacientes con diabetes mellitus tipo 2.
- Testear la aceptación y claridad del material educativo usado para promover cambios de comportamiento.
- Testear la viabilidad de llegar a las metas clínicas propuestas (pérdida de peso y hemoglobina glicosilada).
- Testear la "suficiencia" del monto del incentivo entregado tanto al paciente como a su acompañante, para promover cambios de comportamiento.
- Explorar la sostenibilidad de la participación del acompañante por 9 meses.
- Explorar actividades que podrían hacer al acompañante más efectivo en ayudar al paciente a controlar su diabetes.
- Explorar si el paciente es capaz de llevar un registro diario sobre otros el esfuerzo realizado para manejar su diabetes.
- Evaluar la factibilidad de empaquetar y poner en marcha un ensayo clínico futuro que evalúe el impacto del uso de incentivos monetarios en pacientes con diabetes mellitus tipo 2.

3. METODOLOGIA DEL ESTUDIO

3.1. Diseño del estudio

Estudio piloto que prueba la factibilidad de implementación de tres tipos de intervención con incentivos económicos de manera aleatorizada.

3.2. Lugar de estudio

Consulta externa de los Servicios de Endocrinología del Hospital Nacional Arzobispo Loayza.

3.3. Población y criterios de selección

La población de estudio en general serán pacientes con diabetes mellitus tipo 2 que cumplan los criterios de inclusión y personas de su entorno social que consientan participar en el rol de acompañantes a lo largo de la intervención.

3.3.1 Criterios de inclusión del paciente

Los sujetos serán elegibles si

1. Tienen diagnóstico de diabetes mellitus tipo 2,
2. Tienen entre 18 y 70 años,
3. Reciben tratamiento con Metformina o su tratamiento consiste en cambios en la dieta.
4. Tener un índice de masa corporal entre 25kg/m^2 y 39,9 kg/m^2 (rango que incluye desde sobrepeso hasta obesidad severa),
5. Estar en capacidad de consentir,
6. No estar cumpliendo el rol de acompañante en el marco de este estudio piloto.
7. No tener ceguera, amputaciones o úlceras en los pies ni estar recibiendo diálisis como consecuencia de un mal manejo de la diabetes.

3.3.2 Criterios de inclusión del acompañante

Los acompañantes serán elegidos por los pacientes y además deben:

1. Tener entre 18 y 70 años,
2. No tener ninguna condición física o mental que le impida cumplir el rol de ayudar al participante a bajar de peso,
3. Tener disponibilidad de tiempo y compromiso para apoyar al participante a lograr sus metas de pérdida de peso.
4. Estar en capacidad de consentir.

El consentimiento para participar en el estudio se firmará luego de confirmar la presencia de todos los criterios de inclusión

3.3.3 Criterios de exclusión

- Los sujetos serán excluidos del enrolamiento si tienen cáncer u otra comorbilidad grave.
- Los sujetos serán excluidos si reportan tratamiento farmacológico para bajar de peso o corticoides.
- Los sujetos serán excluidos si están gestando.

3.4. Tamaño de muestra

Setenta y cinco personas (entre pacientes y acompañantes):

- 45 pacientes asignados a tres grupos de intervención, 15 en cada grupo (ver sección 3.5).
- 30 acompañantes: Dado que dos de los grupos requerirán de un acompañante, se enrolarán a 30 personas que acompañen al participante a lo largo de la intervención.

3.5. Intervención

Antes de iniciar la intervención, la educadora en diabetes del estudio (ED) explicará el estudio a los pacientes y responderá sus preguntas. Se obtendrá el consentimiento informado de cada participante y de su acompañante previo al ingreso del estudio. Se tomará información basal que incluirá datos demográficos, nivel socioeconómico, tiempo de enfermedad, tratamiento, complicaciones crónicas propias de la diabetes y comorbilidades, medidas antropométricas (peso, talla, presión arterial), y de laboratorio (hemoglobina glicosilada basal): (Anexo 1: CUESTIONARIO BASAL)

Previamente, la ED contribuirá en la preparación de materiales sobre nutrición y actividad física para personas con diabetes y contará con un rotafolio para su uso en las sesiones educativas (Anexo 2: ROTAFOLIO)

Los pacientes serán aleatorizados a una de las tres intervenciones a ser evaluadas:

Intervención 1: Incentivos Individuales.

1. Los participantes recibirán información sobre el estudio, especificando que recibirán un premio de 150 soles (alrededor de 25% del salario mínimo) en cada reunión si ha logrado bajar un kilo en un periodo de dos semanas. Las reuniones ocurrirán quincenalmente a lo largo de tres meses.
2. Se les hará una evaluación inicial para determinar su peso y se les brindarán los resultados de su Hba1c.
3. Se hará un plan que determine cuántos kilos debe de pesar en cada reunión quincenal (Anexo 3: PLAN)
4. Se les dará educación en nutrición y actividad física.

5. Se le dará un manual con información sobre nutrición y actividad física para personas con diabetes interesadas en bajar de peso, así como consejos y el plan de pérdida de peso por quincena (Anexo 4: MANUAL). La ED sí manejará un plan de pérdida de peso “ideal” para el paciente a lo largo de los 3 meses del estudio.
6. Se les entregará un diario para ingresar su alimentación diaria y si actividad física. La información de este diario será el punto de partida para conversar en cada sesión con la educadora en diabetes sobre las barreras y logros en la implementación del plan de pérdida de peso (Anexo 5: DIARIO).
7. Además de la reunión introductoria, asistirán a siete sesiones de seguimiento (Ver Gráfico 1) y una final en la que se le premiará con el mismo monto (150 soles) si logró bajar la HbA1c en al menos 1%.

Gráfico 1: Ruta del participante

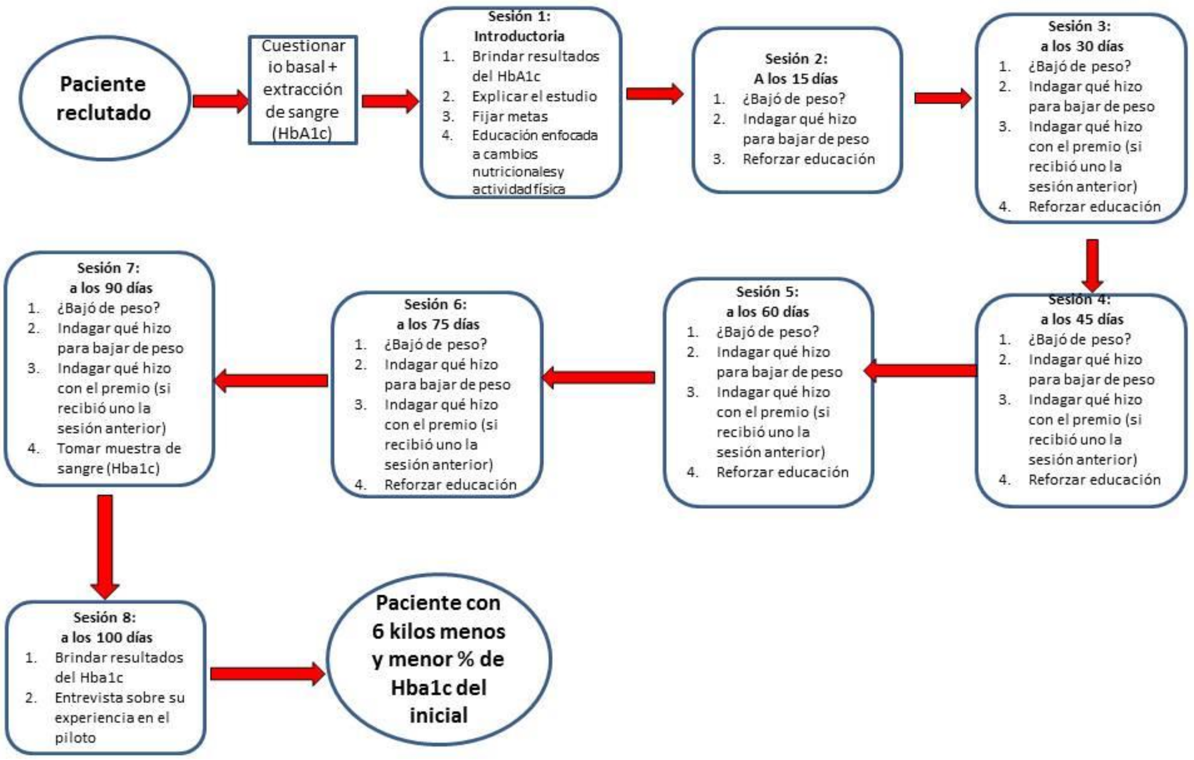

Intervención 2. Incentivos mixtos: Establecidos por investigadores

1. Los participantes y sus acompañantes recibirán información sobre el estudio, especificando que recibirán un premio de 150 soles (alrededor de 25% del salario mínimo) en cada reunión si ha logrado bajar un kilo en un periodo de dos semanas. Las reuniones ocurrirán quincenalmente a lo largo de tres meses. El premio será dividido 50% para el participante y 50% para el acompañante.
2. Se le hará una evaluación inicial al paciente para determinar su peso y se les brindarán los resultados de su Hba1c.
3. Se hará un plan que determine cuántos kilos debe de pesar en cada reunión quincenal (Anexo 3: PLAN).
4. Se les dará educación en nutrición y actividad física de manera oral y/o apoyándose en material audiovisual.
5. Se le dará al acompañante un tríptico que oriente el apoyo que puede brindar (Anexo 5: TRIPITICO)
6. Se les dará al paciente un manual con información sobre nutrición y actividad física para personas con diabetes interesadas en bajar de peso, así como consejos y el plan de pérdida de peso por quincena. La educadora en diabetes sí manejará un plan de pérdida de peso "ideal" para el paciente a lo largo de los 3 meses del estudio (Anexo 4: MANUAL).
7. Se les entregará un diario para ingresar su alimentación éste será el punto de partida para conversar en cada sesión con la educadora en diabetes sobre las barreras y logros en la implementación del plan de pérdida de peso (Anexo 6: DIARIO).
8. Además de la reunión introductoria, asistirán a siete sesiones de seguimiento y una final en la que con el mismo monto (150 soles) si logró bajar la HbA1c en al menos 1%.

Intervención 3. Incentivos mixtos: Establecidos por participantes

1. Los participantes y sus acompañantes recibirán información sobre el estudio, especificando que recibirán un premio de 150 soles (alrededor de 25% del salario mínimo) en cada reunión si ha logrado bajar un kilo en un periodo de dos semanas y si el/la acompañante apoya en el proceso, asistiendo a todas las reuniones quincenales de seguimiento. Las reuniones ocurrirán quincenalmente a lo largo de tres meses. El participante y el acompañante deberán negociar el día de la reunión en que se otorga el premio cómo distribuirán el monto recibido, especificando que el máximo que puede recibir una de las partes es 80% y el mínimo 20% del monto total.
2. Se le hará una evaluación inicial al paciente para determinar su peso y se les brindarán los resultados de su Hba1c.
3. Se hará un plan que determine cuántos kilos debe de pesar en cada reunión quincenal (Anexo 3: PLAN).
4. Se les dará educación en nutrición y actividad física de manera oral y/o apoyándose en material audiovisual.
5. Se le dará al acompañante un tríptico que oriente el apoyo que puede brindar (Anexo 4: TRIPITICO)

6. Se les dará al paciente un manual con información sobre nutrición y actividad física para personas con diabetes interesadas en bajar de peso, así como consejos y el plan de pérdida de peso por quincena. La educadora en diabetes sí manejará un plan de pérdida de peso "ideal" para el paciente a lo largo de los 3 meses del estudio (Anexo 5: MANUAL).
7. Se les entregará un diario para ingresar su alimentación éste será el punto de partida para conversar en cada sesión con la educadora en diabetes sobre las barreras y logros en la implementación del plan de pérdida de peso (Anexo 6: DIARIO).
8. Además de la reunión introductoria, asistirán a siete sesiones de seguimiento y una final en la que se le premiará con el mismo monto (150 soles) si logró bajar la HbA1c en al menos 1%.

3.5 Resultados

Al finalizar de los tres meses se hará una comparación entre los logros en términos de (1) pérdida de peso y (2) hemoglobina glicosilada para luego evaluar a través de un estudio cualitativo si el monto entregado fue el indicado para promover la pérdida de peso, el rol de la educación en diabetes y la valoración del proceso

3.6 Proceso de reclutamiento

3.6.1 Aleatorización y otros procesos

Se seguirán las recomendaciones de la declaración CONSORT [26] en el proceso de aleatorización. Un investigador independiente del proceso del estudio y no involucrado en las actividades de este será el responsable de la aleatorización. Para ello, se usará una aleatorización en bloques de 4 utilizando sobres sellados pre-codificados para ocultar la asignación. Este proceso se llevará a cabo una semana posterior a la firma del consentimiento y de la evaluación inicial.

3.6.2 Procedimientos

Tamizaje

Todos los pacientes que acudan a consulta externa del servicio de endocrinología del Hospital Arzobispo Loayza serán invitados a participar de una evaluación inicial para conocer si son elegibles para participar en el estudio. Esta evaluación consiste en: (1) verificar si el participante tiene sobrepeso, obesidad u obesidad grave (más no obesidad mórbida), (2) confirmar que tiene alguien en su círculo social cercano (familiares o amigos) mayores de edad y saludables que estarían dispuestos a cumplir el rol de "acompañante" que consiste en apoyarlo a lo largo del piloto para lograr sus metas de pérdida de peso, (3) confirmar que su tratamiento para la diabetes consiste en Metformina o cambios en la dieta. Aquellos pacientes que cumplan con los criterios de inclusión, serán invitados a participar en el estudio y una vez que acepten se les sacará una muestra de sangre para documentar sus niveles iniciales de HbA1c.

Fase 1: Línea de base

Se recolectará información inicial que incluye datos demográficos, nivel socioeconómico, antecedentes de importancia como el tiempo desde el diagnóstico, tipo de medicación anti-diabética, estilo de vida (consumo de tabaco, alcohol y actividad física), depresión, presencia de complicaciones de diabetes y comorbilidades (Ver Anexo 1). Se incluirán además medidas antropométricas como el peso, la talla y la presión arterial, y de sangre como la hemoglobina glicosilada (HbA1c).

Fase 2: Intervención

El paciente será contactado a los tres días de la firma del consentimiento y se confirmará su participación. Luego los participantes serán aleatorizados. Aquellos participantes en el grupo de intervención 1 no deberán de traer a su acompañante a ninguna de las reuniones mientras en el caso de la intervención 2 y 3, los acompañantes deberán firmar un compromiso de apoyar al paciente en el plan de pérdida de peso y en compartir el premio.

Posteriormente todos los pacientes seleccionados llenarán el cuestionario de "Inventarios de Personalidad" para recoger datos sobre habilidades no cognitivas. (Ver Anexo 7: INVENTARIOS DE PERSONALIDAD)

Fase 3: Seguimiento de pérdida de peso

El seguimiento estará a cargo de una persona profesional capacitada como Educadora en Diabetes que será entrenada por el personal del proyecto para realizar el seguimiento a los 45 pacientes y sus acompañantes.

Brazo1: Incentivos Individuales

Se pedirá a los participantes que acudan para medir su peso cada 2 semanas y para sacar una muestra de sangre para medir glucosa con un glucómetro. El peso se tomará en ayunas, en la misma balanza y a la misma hora que la visita anterior.

Cada participante asistirá a un total de 8 sesiones (incluyendo la reunión inicial). En las sesiones 2 a 7 la educadora en diabetes los pesará para ver si llegaron a la meta.

- ***Si llegaron a la meta***: Se les premiará y les preguntará sobre las acciones hizo para lograr su objetivo. Adicionalmente se repasarán sugerencias para mejorar su nutrición y manejo de la diabetes. Para ello, usará la información ingresada por los participantes en su diario. Además se les preguntará sobre su actividad física, adherencia al tratamiento y cambios en sus hábitos alimenticios. En caso de haber recibido un premio la sesión previa, se les preguntará sobre el uso que hicieron de ese premio.

- ***Si no llegaron a la meta:*** Se les preguntará sobre las acciones hizo para bajar de peso y los principales retos para implementarlas. Para ello, usará la información ingresada por los participantes en su diario. Se repasarán sugerencias para mejorar su nutrición y manejo de la diabetes. Además se les preguntará sobre su actividad física, adherencia al tratamiento y cambios en sus hábitos alimenticios.
- En cada sesión el paciente llenará un cuestionario de autocuidado de la diabetes (Ver Anexo 8: AUTOCUIDADO DE DIABETES)

En la sesión 7 se le sacará una muestra de sangre para medir su Hba1c. Los resultados se entregaran en la sesión 8 (última sesión) en la que se le premiará si su Hba1c ha bajado al menos 1% desde el inicio del estudio (Ver Anexo 9: DESCRIPCIÓN DETALLADA DE CADA SESIÓN).

Brazo 2: Incentivos Mixtos: Establecido por Investigadores

Se pedirá a los participantes que acudan para medir su peso cada 2 semanas y para sacar una muestra de sangre para medir glucosa con un glucómetro, de ser posible junto con sus acompañantes. Cada pareja (paciente y acompañante) asistirán a un total de 8 sesiones. La entrega del premio es condicional a la presencia del acompañante. En las sesiones 2 a 7 la educadora en diabetes los pesará.

- ***Si llegaron a la meta***: Se premiará al paciente y al acompañante (si éste estuviera presente). Si el acompañante no estuviera en la reunión, la enfermera lo contactará telefónicamente para coordinar la fecha y hora en que puede acercarse a recibir su parte del premio. Se le preguntará al paciente y al acompañante sobre las acciones realizadas para lograr su objetivo. Adicionalmente se repasarán sugerencias para mejorar su nutrición y manejo de la diabetes. Para ello, usará la información ingresada por los participantes en su diario y se les aplicará un cuestionario corto orientado al autoreporte de adherencia, actividad física y cambios alimenticios. En caso de haber recibido un premio la sesión previa, se les preguntará sobre el uso que hicieron de ese premio
- ***Si no llegaron a la meta:*** Se les preguntará sobre las acciones hizo para bajar de peso y los principales retos para implementarlas. Para ello, usará la información ingresada por los participantes en su diario. Se repasarán sugerencias para mejorar su nutrición y manejo de la diabetes. Además se les aplicará un cuestionario corto orientado al autoreporte de adherencia, En la sesión 7 se le sacará una muestra de sangre para medir su Hba1c. Los resultados se entregaran en la sesión 8 (última sesión) en la que se le premiará si su Hba1c ha bajado al menos 1% desde el inicio del estudio y su acompañante los ha acompañado.

Brazo 3: Incentivos Mixtos: Establecido por Participantes

Se pedirá a los participantes que acudan para medir su peso cada 2 semanas y para sacar una muestra de sangre para medir glucosa con un glucómetro, de ser posible junto con sus acompañantes. Cada pareja (paciente y acompañante) asistirán a un total de 8 sesiones. En las sesiones 2 a 7 la educadora en diabetes los pesará.

- ***Si llegaron a la meta***: El paciente y el acompañante deberán negociar en base al esfuerzo percibido por cada una de las partes, la proporción del premio que corresponde a cada uno con la condición de que el máximo porcentaje a recibir es de 80% y el mínimo de 20%. Se premiará al paciente y al acompañante (si éste estuviera presente). Si el acompañante no estuviera en la reunión la negociación ocurrirá telefónicamente y luego, la enfermera lo contactará telefónicamente para coordinar la fecha y hora en que puede acercarse a recibir su parte del premio. Se le preguntará al paciente y al acompañante sobre las acciones realizadas para lograr su objetivo. Adicionalmente se repasarán sugerencias para mejorar su nutrición y manejo de la diabetes. Para ello, usará la información ingresada por los participantes en su diario y se les aplicará un cuestionario corto orientado al autoreporte de adherencia, actividad física y cambios alimenticios.
- ***Si no llegaron a la meta:*** Se les preguntará sobre las acciones hizo para bajar de peso y los principales retos para implementarlas. Para ello, usará la información ingresada por los participantes en su diario. Se repasarán sugerencias para mejorar su nutrición y manejo de la diabetes. Además se les aplicará un cuestionario corto orientado al autoreporte de adherencia, actividad física y cambios alimenticios.
- En la sesión 7 se le sacará una muestra de sangre para medir su Hba1c. Los resultados se entregaran en la sesión 8 (última sesión) en la que se le premiará si su Hba1c ha bajado al menos 1% desde el inicio del estudio y su acompañante los ha acompañado.

Brazo 3: Incentivos Mixtos: Establecido por participantes

Se pedirá a los participantes que acudan para medir su peso cada 2 semanas, de ser posible junto con sus acompañantes. Cada pareja (paciente y acompañante) asistirán a un total de 8 sesiones. En las sesiones 2 a 7 la educadora en diabetes los pesará.

- ***Si llegaron a la meta***: El paciente y el acompañante deberán negociar en base al esfuerzo percibido por cada una de las partes, la proporción del premio que corresponde a cada uno con la condición de que el máximo porcentaje a recibir es de 80% y el mínimo de 20%. Se premiará

al paciente y al acompañante (si éste estuviera presente). Si el acompañante no estuviera en la reunión la negociación ocurrirá telefónicamente y luego, la enfermera lo contactará telefónicamente para coordinar la fecha y hora en que puede acercarse a recibir su parte del premio. Se le preguntará al paciente y al acompañante sobre las acciones realizadas para lograr su objetivo. Adicionalmente se repasarán sugerencias para mejorar su nutrición y manejo de la diabetes. Para ello, usará la información ingresada por los participantes en su diario y se les aplicará un cuestionario corto orientado al autoreporte de adherencia, actividad física y cambios alimenticios. En caso de haber recibido un premio la sesión previa, se les preguntará sobre el uso que hicieron de ese premio

- ***Si no llegaron a la meta:*** Se les preguntará sobre las acciones hizo para bajar de peso y los principales retos para implementarlas. Para ello, usará la información ingresada por los participantes en su diario. Se repasarán sugerencias para mejorar su nutrición y manejo de la diabetes. Además se les aplicará un cuestionario corto orientado al autoreporte de adherencia, actividad física y cambios alimenticios. En caso de haber recibido un premio la sesión previa, se les preguntará sobre el uso que hicieron de ese premio.
- En la sesión 7 se le sacará una muestra de sangre para medir su Hba1c. Los resultados se entregaran en la sesión 8 (última sesión) en la que se le premiará si su Hba1c ha bajado al menos 1% desde el inicio del estudio y su acompañante los ha acompañado.

Fase 3: Seguimiento de experiencias con el estudio

Se usarán entrevistas a profundidad aplicadas a los participantes del estudio. A fin de (a) explorar su opinión sobre el impacto de haber recibido incentivos económicos para perder peso y así mejorar el manejo de su diabetes; (b) explorar su opinión sobre el monto del incentivo, para ver si era considerado mucho, muy poco o razonable, (c) conocer sus opiniones sobre el material educativo recibido (d) conocer (en el brazo 2) el rol que cumplieron los acompañantes y documentar las diversas formas en que apoyaron al paciente a lograr sus metas.

Procedimientos: Las entrevistas serán realizadas por personal apropiadamente capacitado en técnicas cualitativas. Las entrevistas serán audio-grabadas y transcritas textualmente. Se codificarán las transcripciones de acuerdo a temas utilizando ATLAS.ti versión 7 para Windows.

Tamaño de muestra: 30 personas.

- 18 participantes; 6 del brazo de incentivos individuales, 6 del brazo de incentivos mixtos determinados por el investigador y 6 del brazo de incentivos mixtos determinados por los participantes.
- 12 acompañantes: 6 de cada brazo.

3.7. Comunicación de resultados a pacientes

Los resultados de Hba1c, presión arterial y peso serán comunicados y entregados a los participantes a través de la educadora en diabetes del estudio. Se le recomendará a los participantes, de ser necesario, que acuda a su médico de cabecera para una interpretación apropiada de los resultados.

3.8. Cronograma

**Actividades de investigación**	MESES									
	**1**	**2**	**3**	**4**	**5**	**6**	**7**	**8**	**9**	**10**
Preparación de protocolo	x									
Aprobación ética		x	x							
Reclutamiento				x	x					
Intervención					x	x	x	x		
Evaluación cualitativa								x	x	
Transcripción de entrevistas									x	
Análisis de datos						x	x	x		
Redacción de informe final										x

4. PROTECCION DE SUJETOS HUMANOS

4.7. Ética

Este protocolo, cuestionarios y los consentimientos informados – y cualquier modificación subsecuente – será revisado y aprobado por el Comité Institucional de Ética (CIE) del Hospital Nacional Arzobispo Loayza con respecto a su contenido científico y cumplimiento relacionados a investigación en seres humanos.

4.8. Consentimiento Informado

Antes de proveer consentimiento informado, se les dará a los participantes la oportunidad de formular preguntas hasta que comprendan en su totalidad el estudio. El entrevistador firmará la guía del proceso de consentimiento y entregará una copia al entrevistado (Ver Anexo 4).

Todos los participantes tendrán asignados un único código de identificación. Se guardarán los datos en papel y en forma electrónica. La información electrónica será archivada, copiada y asegurada con contraseñas. Los formatos en papel serán almacenados en armarios bajo llave con acceso limitado a individuos específicos. La información personal, incluyendo el nombre del participante, su dirección, fecha de nacimiento y otros potenciales identificadores serán guardados en carpetas protegidas por contraseñas. Solamente el personal del estudio tendrá acceso a esta información.

4.9. Beneficios

Los participantes podrían beneficiarse con los incentivos económicos que reciban y con le educación y los resultados de exámenes médicos entregados durante su participación. Este estudio de investigación subsecuentemente es para que el participante utilice los incentivos para un mejoramiento y control de su enfermedad.

4.10. Riesgos

No se esperan problemas de seguridad importantes en este estudio. Los gastos de transporte, de ser necesarios, serán cubiertos por el proyecto.

4.11. Pago a participantes

Los participantes no recibirán pago alguno por la participación en el presente estudio. Recibirán un incentivo económico por llegar a ciertas metas de pérdida de peso y manejo de la diabetes.

4.12. Confidencialidad

Toda la información relacionada al estudio será almacenada de forma segura. Toda la información de los participantes será almacenada en bases de datos protegidas por contraseñas en computadoras accesibles solo a investigadores del estudio. Todos los reportes, datos del estudio, procesos y formatos administrativos serán identificados solo por un código numérico para mantener la confidencialidad. Toda la información que resulte del presente estudio será tratada con estricta confidencialidad, y solamente los investigadores mencionados en el presente estudio, autoridades regulatorias locales, Comités de Ética, y aquellos que estas designen tendrán acceso a esta información.

Los resultados de este estudio serán presentados por los investigadores a revistas indizadas y revisadas por pares para su publicación.

5. MANEJO DE DATOS Y PLAN DE ANÁLISIS

5.7. Control de calidad y seguridad de los datos

Se propone un programa multifacético para el control de calidad de los datos del estudio que incluye: (1) reuniones y revisiones diarias con los encuestadores de campo para control de calidad así como actualizaciones de entrenamiento, (2) entrada de datos duplicada en una base de datos con capacidad de relacionar ingresos y que posee supervisiones automáticas de la validez de los datos, y (3) una continua revisión de la descripción estadística de datos del estudio por el investigador principal.

Visitas a campo serán realizadas durante el estudio, para asegurar que los requerimientos regulatorios se cumplan. La seguridad de los datos incluye seguridad física de formatos basados en papel y de las computadoras utilizadas para el estudio, así como protección por contraseña y una copia de seguridad de toda la información en las computadoras.

La estrecha cooperación del investigador del estudio, encuestadores de campo, los encargados de los datos, y otros miembros del equipo del estudio será necesaria para seguir el progreso del estudio, responder a las preguntas acerca de la ejecución apropiada del estudio, y abordar otras cuestiones de una manera oportuna.

5.8. Plan de análisis

5.8.1. Línea Basal y Seguimiento

La información recogida será ingresada y tabulada para poder identificar las características de los participantes. Posteriormente los datos serán analizados descriptivamente utilizando medidas de tendencia central, medidas de dispersión, frecuencias absolutas y frecuencias relativas, de acuerdo al tipo de variable.

Para el análisis longitudinal se compararán los tres brazos del estudio (incentivos individuales, incentivos mixtos establecidos por investigadores, incentivos mixtos establecidos por participantes), utilizando como outcomes la diferencia de peso (cada 15 días durante tres meses) y la diferencia en el porcentaje de hemoglobina glicosilada (a los tres meses). Para ello, se utilizarán riesgos relativos con sus intervalos de confianza al 95%.

5.8.2. Estudio Cualitativo: Entrevistas a profundidad. Las entrevistas serán grabadas en mp3, transcritas y luego analizadas usando el software Atlas-ti. Este Software permite codificar las entrevistas a fin de identificar temas comunes, recurrencia de opiniones y detalles explicativos de las respuestas brindadas.

5.9. Cumplimiento del protocolo

El presente estudio se desarrollará a cabalidad de acuerdo al protocolo y a las buenas prácticas clínicas. El protocolo no será enmendado sin tener previamente aprobación escrita por el Comité de Ética. Todas las enmiendas serán enviadas para evaluación a los comités institucionales de ética antes de implementarse, excepto cuando sea necesario proteger la seguridad, derechos o bienestar de los participantes, o para eliminar riesgos inmediatos a los participantes.

5.10. Plan de seguridad de datos

El manejo de datos seguirá estándares de buenas prácticas clínicas. Seguiremos los procedimientos y definiciones estándar para la evaluación y el reporte de eventos adversos. El investigador principal será responsable de reportar todos los eventos adversos que sean observados o reportados durante el estudio. Todo el personal del estudio recibirá instrucciones para detectar y reportar cualquier evento adverso. El investigador principal será responsable de centralizar toda esta información.

6. REFERENCIAS BIBLIOGRAFICAS

1. Lazo MdL, Loza J, de la Cruz C, et al. Quality of metabolic control in ambulatory type 2 diabetes patients under a challenging health system for chronic diseases. Global health, epidemiology and genomics. Submitted 2016.
2. Lerner AG, Bernabe-Ortiz A, Gilman RH, Smeeth L, Miranda JJ. The "Rule of Halves" Does Not Apply in Peru: Awareness, Treatment, and Control of Hypertension and Diabetes in Rural, Urban, and Rural-to-Urban Migrants. Critical pathways in cardiology 2013;12:53-8.
3. The effect of intensive treatment of diabetes on the development and progression of long-term complications in insulin-dependent diabetes mellitus. The Diabetes Control and Complications Trial Research Group. N Engl J Med 1993;329:977-86.
4. Goldman DP, Zheng Y, Girosi F, et al. The benefits of risk factor prevention in Americans aged 51 years and older. Am J Public Health 2009;99:2096-101.
5. Silva H, Hernandez-Hernandez R, Vinueza R, et al. Cardiovascular risk awareness, treatment, and control in urban Latin America. American journal of therapeutics 2010;17:159-66.
6. Gneezy U, Meier S, Rey-Biel P. When and Why Incentives (Don’t) Work to Modify Behavior. . Journal of Economic Perspectives 2011;25:191-210.
7. Halpern SD, Madison KM, Volpp KG. Patients as mercenaries?: the ethics of using financial incentives in the war on unhealthy behaviors. Circ Cardiovasc Qual Outcomes. 2009 Sep2(5):514-6
8. Becker GS, Murphy KM. A Theory of Rational Addiction. Journal of Political Economy 1988;96:675-700
9. Volpp KG, Pauly MV, Loewenstein G, Bangsberg D. P4P4P: an agenda for research on pay-for-performance for patients. Health affairs 2009;28:206-14.
10. O’ Donoghue T, Rabin M. Doing it Now or Later. American Economic Review 1999;89:103-24.
11. O’ Donoghue T, Rabin M. Choice and Procrastination. Quarterly Journal of Economics 2001;116:103-60.
12. Sindelar JL. Paying for performance: the power of incentives over habits. Health economics 2008;17:449-51.
13. Volpp KG, John LK, Troxel AB, Norton L, Fassbender J, Loewenstein G. Financial incentive-based approaches for weight loss: a randomized trial. JAMA 2008;300:2631-7.
14. Volpp KG, Troxel AB, Pauly MV, et al. A randomized, controlled trial of financial incentives for smoking cessation. N Engl J Med 2009;360:699-709.
15. Gneezy U, Rustichini A. Pay enough or don't pay at all. The Quarterly Journal of Economics 2000;115:791-810.

16. Ariely D, Bracha A, Meier S. Doing good or doing well? Image motivation and monetary incentives in behaving prosocially. The American Economic Review, 2009;991.
17. Lacetera N, Macis M. Do all material incentives for pro-social activities backfire? The response to cash and non-cash incentives for blood donations. Journal of Economic Psychology 2010;31:738-48.
18. Kane RL, Johnson PE, Town RJ, et al. Economic Incentives for Preventive Care. Summary, Evidence Report/Technology Assessment: Number 101. AHRQ Publication Number 04E0241, August 2004. Agency for Healthcare Research and Quality, Rockville, MD
19. Lacetera N, Macis M, Slonim R. Will there be blood? Incentives and displacement effects in pro-social behavior. American Economic Journal: Economic Policy 2012;4:186-223.
20. S H, MM K, Y. W. Analyzing compensation methods in manufacturing: Piece rates, time rates or gain-sharing? NBER working paper: #16540 2010.
21. Tsai AG, Wadden TA. Systematic review: an evaluation of major commercial weight loss programs in the United States. Ann Intern Med 2005;142:56-66.
22. Long JA, Jahnle EC, Richardson DM, Loewenstein G, Volpp KG. Peer mentoring and financial incentives to improve glucose control in African American veterans: a randomized trial. Ann Intern Med 2012;156:416-24.
23. Lorincz IS, Lawson BC, Long JA. Provider and patient directed financial incentives to improve care and outcomes for patients with diabetes. Current diabetes reports 2013;13:188-95.
24. Lagarde M, Haines A, Palmer N. The impact of conditional cash transfers on health outcomes and use of health services in low and middle income countries. Cochrane database of systematic reviews 2009:CD008137.
25. Fernald LC, Gertler PJ, Neufeld LM. Role of cash in conditional cash transfer programmes for child health, growth, and development: an analysis of Mexico's Oportunidades. Lancet 2008;371:828-37.
26. Perova E, Vakis R. Welfare impacts of the "Juntos" Program in Peru: Evidence from a non-experimental evaluation The World Bank; 2009.
27. Trujillo A, Fleisher L. Beyond Income, Access and Knowledge: the Education Gradient on Prevention among Diabetics and Hypertensive. Aging and Health 2013.
28. Sloan FA, Padron NA, Platt AC. Preferences, beliefs, and self-management of diabetes. Health services research 2009;44:1068-87.
29. The effect of intensive treatment of diabetes on the development and progression of long-term complications in insulin-dependent diabetes mellitus. The Diabetes Control and Complications Trial Research Group. N Engl J Med 1993;329:977-86.

30. Klonoff D, Schwartz D. An economic of interventions for diabetes. Diabetes Care 23:390-404, 2000.

31. Miranda JJ, Bernabe-Ortiz A, Smeeth L, Gilman RH, Checkley W. Addressing geographical variation in the progression of non-communicable diseases in Peru: the CRONICAS cohort study protocol. *BMJ open* 2012; **2**(1): e000610.

32. Chobanian, A. V., G. L. Bakris, H. R. Black, W. C. Cushman, L. A. Green, J. L. Izzo, Jr., D. W. Jones, B. J. Materson, S. Oparil, J. T. Wright, Jr., E. J. Roccella, L. National Heart, D. E. Blood Institute Joint National Committee on Prevention, P. Treatment of High Blood and C. National High Blood Pressure Education Program Coordinating (2003). "The Seventh Report of the Joint National Committee on Prevention, Detection, Evaluation, and Treatment of High Blood Pressure: the JNC 7 report." JAMA 289(19): 2560-2572.

33. Flores Juan C, Alvo M, Borja H, Morales J, Vega J, Zúñiga C. Enfermedad renal crónica: Clasificación, identificación, manejo y complicaciones. Rev. médica Chile [Revista en Internet]. 2009 Enero [Citado 2015 Feb 17]; 137(1): 137-177.

34. Bloom S, Till S, Sonksen P, Smith S: Use of biothesiometer to measure individual vibration perception thresholds and their variation in 519 non-diabetic subjects. *Br Med J* 288:1793–1795,1984

35. Young MJ, Breddy JL, Veves A, Boulton AJ: The prediction of neuropathic foot ulceration using vibration perception thresholds: a prospective study. *Diabetes Care* 17:557–560, 1994

36. "PanOptic," www.welchallyn. comlpromotions/PanOptic/default.htm.

37. G. Bradski, "The OpenCV Library," Dr. Dobb's Journal of Software Tools, 2000.

38. Schulz KF, Altman DG, Moher D, Group C. CONSORT 2010 statement: updated guidelines for reporting parallel group randomised trials. PLoS Med 2010;7:e1000251.
