# Supplementary material for: The effect of individual and mixed rewards on diabetes management: A feasibility randomized controlled trial
Source: Wellcome Open Res. 2019 Feb 5;3:139. Originally published 2018 Oct 31. [Version 3] doi: 10.12688/wellcomeopenres.14824.3 (PMC6325609; doi:10.12688/wellcomeopenres.14824.3)
Supplement: Supplementary file 4 [file wellcomeopenres-3-16478-s0003.tgz › c853da35-9d26-4456-b5ce-0712e846d356_Supplementary_File_4_18oct2018.docx]

# Supplementary File 4

## Supplementary Table 1. Intermediate outcomes at baseline and 3-month results, by study arms

|  | Arm 1  Individual | Arm 2  Mixed  Altruism | Arm 3  Mixed  Cooperation | Arm 2  vs.  Arm 1  (p-value) | Arm 3  vs.  Arm 1  (p-value) |
| --- | --- | --- | --- | --- | --- |
| **Intermediate outcomes** |  |  |  |  |  |
| Self-management |  |  |  |  |  |
| Baseline | 12.9 ± 7.3 | 13.1 ± 6.6 | 11.7 ± 6.6 | 0.99 | 0.99 |
| 3 months | 22.0 ± 5.7 | 22.0 ± 5.7 | 19.5 ± 5.0 | 0.99 | 0.47 |
| Δ (3-mo vs baseline) | **9.5 ± 6.4** | **9.8 ± 7.4** | 6.8 ± 9.0 | 0.99 | 0.99 |
| Diet |  |  |  |  |  |
| Baseline | 8.3 ± 4.8 | 8.8 ± 3.5 | 7.6 ± 5.7 | 0.99 | 0.99 |
| 3 months | 16.9 ± 3.0 | 16.8 ± 3.9 | 14.2 ± 5.2 | 0.99 | 0.30 |
| Δ (3-mo vs baseline) | **8.4 ± 5.9** | **7.5 ± 4.9** | **5.4 ± 5.3** | 0.99 | 0.54 |
| Physical activity |  |  |  |  |  |
| Baseline | 4.7 ± 4.0 | 4.2 ± 4.2 | 4.1 ± 3.3 | 0.99 | 0.99 |
| 3 months | 7.1 ± 3.6 | 7.3 ± 3.9 | 5.6 ± 2.7 | 0.99 | 0.99 |
| Δ (3-mo vs baseline) | 1.5 ± 5.4 | **3.8 ± 3.4** | 1.9 ± 4.8 | 0.82 | 0.99 |

Values in bold are those significant (p<0.05) when comparing difference between 3-month and baseline assessment (within each arm).

## Supplementary Table 2. Variation over time of continuous variables

|  | Weight (Kg) | BMI (Kg/m^2^) | Diabetes self-management (points) |
| --- | --- | --- | --- |
| **Study Arm 1** |  |  |  |
| Baseline | **76.9**  **(p<0.001)** | **33.1**  **(p<0.001)** | **12.9**  **(p<0.001)** |
| Δ 2 weeks vs baseline | **-1.0**  **(p=0.01)** | **-0.5**  **(p<0.001)** | **5.5**  **(p = 0.001)** |
| Δ 4 weeks vs baseline | **-1.4**  **(p<0.001)** | **-0.6**  **(p<0.001)** | **8.3**  **(p<0.001)** |
| Δ 6 weeks vs baseline | **-1.4**  **(p<0.001)** | **-0.6**  **(p<0.001)** | **7.7**  **(p<0.001)** |
| Δ 8 weeks vs baseline | **-1.9**  **(p<0.001)** | **-0.8**  **(p<0.001)** | **11.7**  **(p<0.001)** |
| Δ 10 weeks vs baseline | **-2.2**  **(p<0.001)** | **-0.9**  **(p<0.001)** | **11.0**  **(p<0.001)** |
| Δ 12 weeks vs baseline | **-2.8**  **(p<0.001)** | **-1.2**  **(p<0.001)** | **9.3**  **(p<0.001)** |
| **Study Arm 2** |  |  |  |
| Baseline | 84.5  (p = 0.17) | 34.6  (p = 0.41) | 13.0  (p = 0.99) |
| Δ 2 weeks vs baseline | 0.6  (p = 0.29) | 0.2  (p = 0.13) | 1.6  (p = 0.52) |
| Δ 4 weeks vs baseline | 0.6  (p = 0.30) | 0.3  (p = 0.13) | 0.8  (p = 0.74) |
| Δ 6 weeks vs baseline | 1.0  (p = 0.1) | 0.4  (p = 0.13) | 1.2  (p = 0.63) |
| Δ 8 weeks vs baseline | 0.9  (p = 0.14) | 0.4  (p = 0.21) | -2.4  (p = 0.37) |
| Δ 10 weeks vs baseline | **1.4**  **(p = 0.02)** | **0.6**  **(p = 0.05)** | -0.2  (p = 0.93) |
| Δ 12 weeks vs baseline | **2.3**  **(p<0.001)** | **0.9**  **(p = 0.004)** | -0.1  (p = 0.99) |
| **Study Arm 3** |  |  |  |
| Baseline | 85.2  (p = 0.14) | 36.1  (p = 0.25) | 11.6  (p = 0.55) |
| Δ 2 weeks vs baseline | 0.5  (p = 0.37) | 0.2  (p = 0.33) | 1.2  (p = 0.66) |
| Δ 4 weeks vs baseline | 0.5  (p = 0.40) | 0.2  (p = 0.40) | 0.9  (p = 0.72) |
| Δ 6 weeks vs baseline | **1.2**  **(p = 0.05)** | **0.5**  **(p = 0.05)** | 0.5  (p = 0.86) |
| Δ 8 weeks vs baseline | **1.6**  **(p = 0.008)** | **0.6**  **(p = 0.09)** | -2.5  (p = 0.34) |
| Δ 10 weeks vs baseline | **1.9**  **(p = 0.003)** | **0.7**  **(p = 0.06)** | -3.1  (p = 0.25) |
| Δ 12 weeks vs baseline | **2.4**  **(p < 0.001)** | **1.0**  **(p = 0.01)** | -2.6  (p = 0.39) |

Values in bold are those significant (p<0.05).
