# Supplementary material for: The effect of individual and mixed rewards on diabetes management: A feasibility randomized controlled trial
Source: Wellcome Open Res. 2019 Feb 5;3:139. Originally published 2018 Oct 31. [Version 3] doi: 10.12688/wellcomeopenres.14824.3 (PMC6325609; doi:10.12688/wellcomeopenres.14824.3)
Supplement: Peer review history

# Peer review: Referee response for version 2

**DOI:** 10.21956/wellcomeopenres.16291.r34300

**Published:** 2019-01-04

**Authors:** Rachel Nugent

**Affiliations:** 1 Research Triangle Institute (RTI) International, Durham, NC, USA

I find the responses and revisions to be acceptable. The paper presents interesting preliminary results which suggest clear future needed research. I recommend that the study be very clearly presented as intended to generate hypotheses. Thank you for the opportunity to review.

I have read this submission. I believe that I have an appropriate level of expertise to confirm that it is of an acceptable scientific standard.

# Response

**Published:** 2019-01-17

**Authors:** Jaime Miranda

**Affiliations:** Universidad Peruana Cayetano Heredia, Peru

Many thanks indedd for finding our responses and revisions to be acceptable. We appreciate your comment that "the paper presents interesting preliminary results which suggest clear future needed research" and concur with your recommendation "that the study [is] intended to generate hypotheses."

# Peer review: Referee response for version 2

**DOI:** 10.21956/wellcomeopenres.16291.r34302

**Published:** 2019-01-02

**Authors:** Gustavo Angeles

**Affiliations:** 1 Department of Maternal and Child Health, University of North Carolina at Chapel Hill, Chapel Hill, NC, USA

This is an interesting paper about the important topic of providing incentives for healthy diabetes management. The topic is clearly relevant. Some comments:

 1. Intro. Par 3. It will be useful for the reader to have a brief summary of “the limitations of individual-based incentivization strategies”. It will help with the motivation for mixed incentives.

 2. It is not clear why formal testing for comparison of baseline data was not pursued. Authors should elaborate on the reasons. Randomization is expected to provide similar groups, but only if the sample size is “large” and, in practice, even a well conducted randomization procedure could produce unbalanced groups, just by chance. One of the reasons for conducting a baseline is precisely to check that balance among intervention arms was achieved.

 3. 783 participants were screened and according to Figure 1, 705 were excluded. That gives 78 individuals eligible for the study. However, only 54 were randomized. What did happen with the other 24 individuals not included in the study? How come the 54 included in the study were selected out of the 78 eligible?

 4. Given that 18.5% of the baseline group was lost to follow-up, was there any analysis of potential selective attrition (or selective lost-to-follow-up)?

 5. Table 2.Could the authors elaborate on how the results presented in the table were obtained? In particular, taking the difference of the 3 months and baseline means does not match the difference presented in the table. For instance, for the primary outcome, Arm 1, 6.9 – 8.5 = -1.6, however, the table shows -1.4.For Arm 2, 7.2 – 7.9 = -0.7, but the table shows -0.9. There are other cases like those.

 6. Also, in Table 2, I assume the authors present the bounds of the 95% confidence intervals, and they indicate that values in bold are significant at the 5% level.But, how is it significant the difference for Arm 3, primary outcome, if the confidence interval is -1.1 +- 1.6, which means the CI is [-2.7, 0.5]? Similar question for Arm 2, which has a CI of -0.9 +- 1.2 or [-2.1, 0.3]. In both cases, the CI contains the value of zero. There are other cases like those in the tables.

 7. For this being called a feasibility study this referee was expecting more content on the practical aspects of the design and implementation of the intervention. Issues of acceptability by the intended target population, practicalities and challenges of implementation, integration of the intervention with the existing service infrastructure and other aspects usually seen in feasibility studies are barely presented. There is a mention in the Discussion section about the study providing pragmatic insights on an important topic of research, but they are not elaborated at length in the paper. This study is more a pilot evaluation of an intervention with a small sample of analysis. That is fine as pilots are valuable in themselves, and this referee finds this study useful. But it is interesting the selection of words by the authors.

I have read this submission. I believe that I have an appropriate level of expertise to confirm that it is of an acceptable scientific standard.

# Response

**Published:** 2019-01-30

**Authors:** Jaime Miranda

**Affiliations:** Universidad Peruana Cayetano Heredia, Peru

**Comment #1**. Intro. Par 3. It will be useful for the reader to have a brief summary of “the limitations of individual-based incentivization strategies”. It will help with the motivation for mixed incentives.

**Response #1**. Indeed, those limitations are important and here, for the benefit of the reader given the open access platform, we provide some additional information in that regard.

 “ *Individual cash rewards appear to be more effective in the case of standardized activities or in cases when it is relatively easy to observe effort, to monitor outcomes, and when changes are driven by short-term behaviors and goals. In the same vein, individual cash rewards may be less effective in situations where i) highly complex tasks are needed; ii) in activities and target* *behaviours* *that require dealing* *within* *social norms, trust, and reputation; iii) in activities where it is difficult to clearly observe effort and thus change in outcomes; and iv) in activities that involve long term change.[1, 2]*”

**Comment #2**. It is not clear why formal testing for comparison of baseline data was not pursued. Authors should elaborate on the reasons. Randomization is expected to provide similar groups, but only if the sample size is “large” and, in practice, even a well conducted randomization procedure could produce unbalanced groups, just by chance. One of the reasons for conducting a baseline is precisely to check that balance among intervention arms was achieved.

**Response #2**. That is correct, and, in the same vein, there are many other mis-uses of baseline clinical trial data.[3, 4] Ours was a feasibility study, and therefore we preferred not to calculate such testings, as stated in our Method’s Analytical Methods: “ *Following trial recommendations, no formal statistical testing for comparison of baseline data was pursued*.”

**Comment #3**. 783 participants were screened and according to Figure 1, 705 were excluded. That gives 78 individuals eligible for the study. However, only 54 were randomized. What did happen with the other 24 individuals not included in the study? How come the 54 included in the study were selected out of the 78 eligible?

**Response #3**. Thank you for noticing this, it was an omission. Those 24 correspond to individuals who did not show up to the baseline visit, who are different from the other 84 who cancelled the baseline appointment visit. We have updated Figure 1 to include these 24 subjects.

**Comment #4**. Given that 18.5% of the baseline group was lost to follow-up, was there any analysis of potential selective attrition (or selective lost-to-follow-up)?

**Response #4**. That overall figure was correct, and the number of individuals lost to follow-up was similar in each study arm. Our manuscript reports results of a formative research and provides insights to better inform a future large-scale study. Any future handling of this data, or newer analysis, should carefully account for this potential loss to follow-up, and we thank the reviewer for this recommendation.

**Comment #5**. Table 2. Could the authors elaborate on how the results presented in the table were obtained? In particular, taking the difference of the 3 months and baseline means does not match the difference presented in the table. For instance, for the primary outcome, Arm 1, 6.9 – 8.5 = -1.6, however, the table shows -1.4.For Arm 2, 7.2 – 7.9 = -0.7, but the table shows -0.9. There are other cases like those.

**Response #5**. The reviewer is correct in noticing this. The discrepancy observed is explained because the difference is calculated only among those with completed baseline and 3-month data, therefore the number of subjects is not the same as the number of subjects enrolled at baseline.

**Comment #6**. Also, in Table 2, I assume the authors present the bounds of the 95% confidence intervals, and they indicate that values in bold are significant at the 5% level.But, how is it significant the difference for Arm 3, primary outcome, if the confidence interval is -1.1 +- 1.6, which means the CI is [-2.7, 0.5]? Similar question for Arm 2, which has a CI of -0.9 +- 1.2 or [-2.1, 0.3]. In both cases, the CI contains the value of zero. There are other cases like those in the tables.

**Response #6**. We understand the difficulty in understanding this table with multiple estimates and comparisons. To guide the reading of this table, we can summarise it into 3 types of information.

 First, point estimates, the three columns, one for each study Arm, show means (SDs).

 Second, the row “Δ (3-mo vs baseline)” does not show the bounds of 95% CIs, but means (SDs). Also, if bold, it indicates that this comparison is significant, i.e. different from zero, and such comparison was obtained using paired t-tests.

 Third, the last two columns show p-value calculations for differences between two study arms, and these were obtained using t-tests.

**Comment #7**. For this being called a feasibility study this referee was expecting more content on the practical aspects of the design and implementation of the intervention. Issues of acceptability by the intended target population, practicalities and challenges of implementation, integration of the intervention with the existing service infrastructure and other aspects usually seen in feasibility studies are barely presented. There is a mention in the Discussion section about the study providing pragmatic insights on an important topic of research, but they are not elaborated at length in the paper. This study is more a pilot evaluation of an intervention with a small sample of analysis. That is fine as pilots are valuable in themselves, and this referee finds this study useful. But it is interesting the selection of words by the authors.

**Response #7**. The reviewer signals some key aspects of any feasibility study. The rationale for using the term feasibility in this work was informed by a combination of issues related to the fieldwork, to packaging and deploying the intervention, and the experience of the support partner. In terms of fieldwork, we document the ability to approach, identify eligible individuals, as well as to recruit, enroll, and follow-up sufficient number of patients and partners (see study’s flowchart). Key aspects of the intervention include the capacity to deliver the intervention, an intense intervention with tailoring features, and to have a separate team to measure outcomes, expressed in the results reported. Acceptability of the intervention and challenges of the implementation were also captured in the post-study interviews, and described in the Results’ section under “Partner Support”. A combination of all of these inputs will inform whether the intervention merits revision according to its intensity and duration, paired with the team needed, e.g. number of diabetes educator and fieldwork evaluators, and the need to consider multiple sites, in any future larger study, translating into costs and time to execute such study. Much of this text has now been added to the discussion. In addition, we have also added in the introduction the following statement: “ *Overall, this study was framed as a feasibility study designed to generate hypotheses to be tested in future larger studies and to explore the feasibility of conducting complex interventions for diabetes management in terms of fieldwork enrolment and follow-up, delivery* *and* *uptake of the intervention, and challenges of having a partner support.*”

**References**

 1. Gneezy U, Meier S, Rey-Biel P. When and Why Incentives (Don’t) Work to Modify Behavior. J Econ Perspect. 2011;25:191–210.

 2. Ariely D, Bracha A, Meier S. Doing Good or Doing Well? Image Motivation and Monetary Incentives in Behaving Prosocially. Am Econ Rev. 2009;99:544–55.

 3. Assmann SF, Pocock SJ, Enos LE, Kasten LE. Subgroup analysis and other (mis)uses of baseline data in clinical trials. Lancet. 2000;355:1064–9.

 4. Altman DG, Doré CJ. Randomisation and baseline comparisons in clinical trials. Lancet. 1990;335:149–53.

# Peer review: Referee response for version 1

**DOI:** 10.21956/wellcomeopenres.16153.r34164

**Published:** 2018-11-12

**Authors:** Rachel Nugent

**Affiliations:** 1 Research Triangle Institute (RTI) International, Durham, NC, USA

The article addresses the important topic of diabetes management which has been especially neglected in low-resource settings. The study described is interesting and novel in using a monetary incentive to encourage behavioral change that improves diabetes-related health outcomes. As formative research, the work can be used to generate hypotheses and can be replicated. It offers novel work on incentive-setting that can be valuable in many different study settings.

 There are some concerns about the methods and conclusions that I suggest the authors consider.

 It is not clear why there is no control group. I am interested in whether incentives for preventive care improve upon the status quo, which is no incentive. If that question has already been thoroughly explored, then that previous research should be described.

 It would be interesting to know more about the patients' partners, such as their own health status. There may be an association between the diabetic patient's success on the outcomes and the partner's own health. I suggest the same questions be asked of participant's partners as the participant survey.

 There are large differences across the three arms on many of the baseline characteristics because the samples are small. For instance, the level of education is quite different across groups. Although the groups are randomized, demographic variables can influence the stated outcomes. An example is that incentive-related behavior is related to income and education.  

 I suggest more discussion of the findings. The point is made that the study demonstrates feasibility of larger studies, but I remain to be convinced that this intensive intervention, both in educator time and monitoring time, is practical at a large scale except in a trial setting. Also please discuss further the behavioral aspects of the results, including spelling out what lessons were obtained for the practicalities of larger studies, as suggested on page 9 and 10.

 The lack of additional benefit from the caregiver participation is a major finding, and a counter-intuitive one as I would have expected stronger results due to the patient's commitment to those family and friends. 

 Minor:

 There is no mention of how the 29 individuals were selected for in-depth interviews.

 The paragraph mentioning income effects is unclear.

I have read this submission. I believe that I have an appropriate level of expertise to state that I do not consider it to be of an acceptable scientific standard, for reasons outlined above.

# Response

**Published:** 2018-11-16

**Authors:** Jaime Miranda

**Affiliations:** Universidad Peruana Cayetano Heredia, Peru

Responses to Reviewer

**Comment #1**. The article addresses the important topic of diabetes management which has been especially neglected in low-resource settings. The study described is interesting and novel in using a monetary incentive to encourage behavioral change that improves diabetes-related health outcomes. As formative research, the work can be used to generate hypotheses and can be replicated. It offers novel work on incentive-setting that can be valuable in many different study settings.

There are some concerns about the methods and conclusions that I suggest the authors consider.

**Response #1**. Many thanks for your advice and feedback. The reviewer is right in approaching this report as " ***formative research***".

**Comment #2**. It is not clear why there is no control group. I am interested in whether incentives for preventive care improve upon the status quo, which is no incentive. If that question has already been thoroughly explored, then that previous research should be described.

**Response #2**. Indeed, this work may appear as incomplete at this stage, and this can be explained. Rather than embarking into multiple aspects of a complete study, the research team made an open decision to focus this feasibility study in developing, packaging and delivering a reward-based strategy. For example, we had to decide against having a control group because, as a feasibility approach, the study will not be powered to test comparisons between the intervention vs. control groups. Instead, we decided to take one further step and accommodate, from an exploratory angle, groups based on altruism and cooperation. By completing this feasibility study, we demonstrate the acceptability of the intervention to the study participants, the capacity to deliver these interventions, and the readiness to embark into a larger full/complete randomized controlled study.

**Comment #3**. It would be interesting to know more about the patients' partners, such as their own health status. There may be an association between the diabetic patient's success on the outcomes and the partner's own health. I suggest the same questions be asked of participant's partners as the participant survey.

**Response #3**. This is a very good recommendation and one that we can accommodate in a future larger study. Thank you for this suggestion!

**Comment #4**. There are large differences across the three arms on many of the baseline characteristics because the samples are small. For instance, the level of education is quite different across groups. Although the groups are randomized, demographic variables can influence the stated outcomes. An example is that incentive-related behavior is related to income and education.  

**Response #4**. Correct, randomization at baseline do show imbalances, and this is because the study’s sample size is too small as pointed out. We are cautious with our interpretation. Indeed, in the discussion, we express the following: “ *Our pilot was not designed to formally test comparisons between study arms [...]. We express caution with the interpretation of results as it cannot be ascertained whether the changes in primary or secondary outcomes are due to the effect of the intervention in a given study arm. If anything, changes were observed in reducing HbA1c levels and important lessons were obtained for the practicalities of conducting larger studies using mixed incentives and enabling activities between patients, companion supports and diabetes educators*.”

**Comment #5**. I suggest more discussion of the findings. The point is made that the study demonstrates the feasibility of larger studies, but I remain to be convinced that this intensive intervention, both in educator time and monitoring time, is practical at a large scale except in a trial setting. Also please discuss further the behavioral aspects of the results, including spelling out what lessons were obtained for the practicalities of larger studies, as suggested on page 9 and 10.

**Response #5**. We agree that this is an intensive intervention, yet its practicality will need to weighted against its effectiveness (which remains to be seen) and the large societal costs related to diabetes. Two arguments can be put forward. First, today’s status quo is quite shocking, with almost half of UK and US adults with diabetes as well as more than 90% of Peruvian patients not meeting the recommended goals for diabetes care. [1–3] Something needs to be done. Second, there are recent promising results arising from the DIRECT study, conducted in the primary care network, which has shown that diabetes remission can be achieved through intense 12-month weight loss management program. [4, 5] Whilst the intensity of the intervention is a key factor to consider, there are other factors as well. If weight-loss intense strategies can direct towards people engaging with better diet and lifestyles for longer periods, a lesser use of pharmacological medications with its associated risks, and even diabetes remission, then the scenario will be different. We will be able to complement this evidence, in a future study, by studying the effect of individual and group-based rewards. We have expanded the discussion with these reflections.

**Comment #6**. The lack of additional benefit from the caregiver participation is a major finding, and a counter-intuitive one as I would have expected stronger results due to the patient's commitment to those family and friends.

**Response #6**. Unfortunately, it is premature to reach to such a conclusion, we need a full larger study to answer such a question.

**Comment #7**. Minor: There is no mention of how the 29 individuals were selected for in-depth interviews.

**Response #7**. We aimed to select 6 participants per study arm (18 in total), and 6 partners per study arm (12 in total), and we managed to interview 29/30 out of the planned target. Within each 6, we selected 3 who “performed well during the intervention”, defined as those who were able to lose 3Kg or more, and 3 others who did not. This detail has been added to the manuscript.

**Comment #8**. Minor: The paragraph mentioning income effects is unclear.

**Response #8**. We have edited this paragraph, which now reads, “ *We kept the size of the reward equal between the treatment groups to avoid an income effect at the household level, i.e. the reward size was the same across all study arms regardless of the involvement of the partner support*.” To explain further, a participant in the study Arm 1 would have received a pre-specified amount for each target accomplished, whereas in study Arm 3 the same amount would have to be shared between the participant and his partner. In the latter scenario, this approach implies a lower reward per each individual (the patient and his partner). The alternative scenario would have been to offer the same amount for each individual across irrespective of their study Arm allocation, however, the household’s of participants assigned to patient+partner would have received twice as much as those in the individual-only Arm, generating an income effect within the household. This is what we are trying to explain in this paragraph. If this is still confusing, we are willing to hear advice on how best to phrase this information.

**References**

1. Ali MK, Bullard KM, Saaddine JB, Cowie CC, Imperatore G, Gregg EW. Achievement of goals in U.S. diabetes care, 1999-2010. N Engl J Med. 2013;368:1613–24.

2. Huayanay-Espinoza IE, Guerra-Castañon F, Lazo-Porras M, Castaneda-Guarderas A, Thomas NJ, Garcia-Guarniz A-L, et al. Metabolic control in patients with type 2 diabetes mellitus in a public hospital in Peru: a cross-sectional study in a low-middle income country. PeerJ. 2016;4:e2577.

3. Khunti K, Gadsby R, Millett C, Majeed A, Davies M. Quality of diabetes care in the UK: comparison of published quality-of-care reports with results of the Quality and Outcomes Framework for Diabetes. Diabet Med. 2007;24:1436–41.

4. Lean ME, Leslie WS, Barnes AC, Brosnahan N, Thom G, McCombie L, et al. Primary care-led weight management for remission of type 2 diabetes (DiRECT): an open-label, cluster-randomised trial. Lancet. 2018;391:541–51.

5. Leslie WS, Ford I, Sattar N, Hollingsworth KG, Adamson A, Sniehotta FF, et al. The Diabetes Remission Clinical Trial (DiRECT): protocol for a cluster randomised trial. BMC Fam Pract. 2016;17:20.

# Response

**Published:** 2019-01-03

**Authors:** Rachel Nugent

**Affiliations:** RTI International, USA

Thank you for the clear and acceptable responses.
